# Hornerin contains a Linked Series of Ribosome-Targeting Peptide Antibiotics

**DOI:** 10.1038/s41598-018-34467-8

**Published:** 2018-11-01

**Authors:** Ulrich Gerstel, Ties Latendorf, Joachim Bartels, Alexander Becker, Andreas Tholey, Jens-Michael Schröder

**Affiliations:** 1 Department of Dermatology, University-Hospital Schleswig-Holstein, Campus Kiel, Kiel, Germany; 2 Institute for Experimental Medicine – AG Systematic Proteomics & Bioanalytics, Kiel University (CAU), Kiel, Germany

## Abstract

Cationic intrinsically disordered antimicrobial peptides (CIDAMPs) belong to a novel class of epithelial peptide antibiotics with microbicidal activity against various pathogens, including *Pseudomonas aeruginosa*, *Escherichia coli*, *Staphylococcus aureus* and *Candida albicans*. Here we show that treatment of distinct bacteria with different hornerin (HRNR)-derived CIDAMPs cause formation of unique cytoplasmic protein aggregates, suggesting a common intracellular mode of action. We further found that, unlike most amphipathic antimicrobial peptides, HRNR traverses bacterial membranes energy-dependently and accumulates within the cytoplasm. Strikingly, certain structurally different, HRNR-based CIDAMPs were found to bind to an identical panel of distinct bacterial ribosomal proteins, thereby manifesting features of several known classes of antibiotics. This may cause the formation of aberrant proteins and toxic protein aggregates in HRNR-treated pathogens which eventually may induce its death. Our study reveals evidence that structurally distinct CIDAMPs of an abundant body surface protein simultaneously target multiple sites of the bacterial protein synthesis machinery.

## Introduction

By unknown reasons, healthy human skin is remarkably resistant towards infection by *Pseudomonas* (*P*.) *aeruginosa*, an environmental opportunistic pathogen widespread in water and soil. In an effort to get insight into this unusual natural resistance, we recently identified in heel stratum corneum-extracts peptide fragments of the epidermal intrinsically disordered S100-fused-type 254 kDa protein hornerin (HRNR) as potent, at skin-relevant environmental conditions *P*. *aeruginosa*-cidal antimicrobial peptides (AMPs) (TL, UG, Zhihong Wu, JB, AB, AT and JMS, Sci. Rep., in revision). HRNR is highly expressed in healthy skin^1^ where it can form nanofiber scaffolds in stratum corneum^2^. It consists of 95% of glycine- and serine-rich highly cationic repeat domains and it is present in skin as a complex mixture of multimeric polypeptide fragments^1,3^. These repeat domains were identified as linked series of “cationic intrinsically disordered antimicrobial peptides, CIDAMPs”, microbicidal peptides rich in disorder-promoting polar amino acids like Gly/Ser/Thr/Gln/His and low in order-promoting, hydrophobic AA like Leu/Ile/Val/Asp/Tyr/Phe/Trp (TL, UG, Zhihong Wu, JB, AB, AT and JMS, Sci. Rep., in revision). Strikingly, quantitative proteome analyses identified HRNR as highly abundant protein in humans^4^. Here, mainly epithelial cell types revealed highest HRNR-abundance in barrier organs (kidney, skin, lung, vulva, colon, rectum, urinary bladder, uterine cervix, placenta). Further, immune privileged organs and organs where vital structures need to be protected from the potentially damaging effects of an inflammatory immune response (eye, brain, central nervous system, female gonads, teeth as well as the heart) revealed a similar HRNR abundance^4^. This suggests that HRNR-derived CIDAMPs are important innate defense effector peptides, acting at the outermost surface of healthy barrier organs as disinfectants, possibly helping to keep the surface of skin and mucosa free of infection by commensals as well as environmental microbes (TL, UG, Zhihong Wu, JB, AB, AT and JMS, Sci. Rep., in revision).

A panel of recombinantly and by chemical synthesis generated HRNR-polypeptide-fragments revealed to be microbicidal AMPs with higher potency than most amphipathic AMPs, targeting *P*. *aeruginosa*, *Escherichia* (*E*.) *coli*, *Candida* (*C*.) *albicans* and, to a lesser degree, also *Staphylococcus* (*S*.) *aureus* (TL, UG, Zhihong Wu, JB, AB, AT and JMS, Sci. Rep., in revision). It is therefore aimed to explore, why and how HRNR-derived CIDAMPs are able to exert its potent microbicidal activity.

## Ultrastructural Analyses Reveal a Unique Morphology of CIDAMP-Treated Micro-organisms

Most known antimicrobial proteins and peptides possess diverse secondary structures with an amphipathic surface in hydrophobic environments^5^. These AMPs are largely targeting the bacterial membrane, but can have also multiple other modes of action that differ from those of conventional antibiotics^5^. Disturbance of bacterial membrane integrity can directly or indirectly cause metabolic dysfunction and cell death, besides pore formation *per se*^6^.

Observing alterations in bacterial membrane integrity by transmission electron microscopy (TEM) can give first hints on the detailed mechanisms of cell death at lethal AMP concentrations.

Having demonstrated that HRNR-polypeptide-fragments are bactericidal antimicrobials, killing also the yeast *C*. *albicans*, we wondered whether these peptides actively destroy microbial cells by direct membrane effects or whether they rather act indirectly via an intracellular target. To achieve this, selected strains of *E*. *coli*, *P*. *aeruginosa*, *S*. *aureus* and *C*. *albicans* were challenged with distinct HRNR-derived CIDAMPs, rHRNR_2591–2684_, rSUMO3-HRNR_2591–2684_, HRNR_1132–1143_ (HR2-8), HRNR_2606–2628_ (HR1-11) and HRNR_2656–2677_ (HR1-18), respectively, and then imaged by TEM.

**Figure 1:**
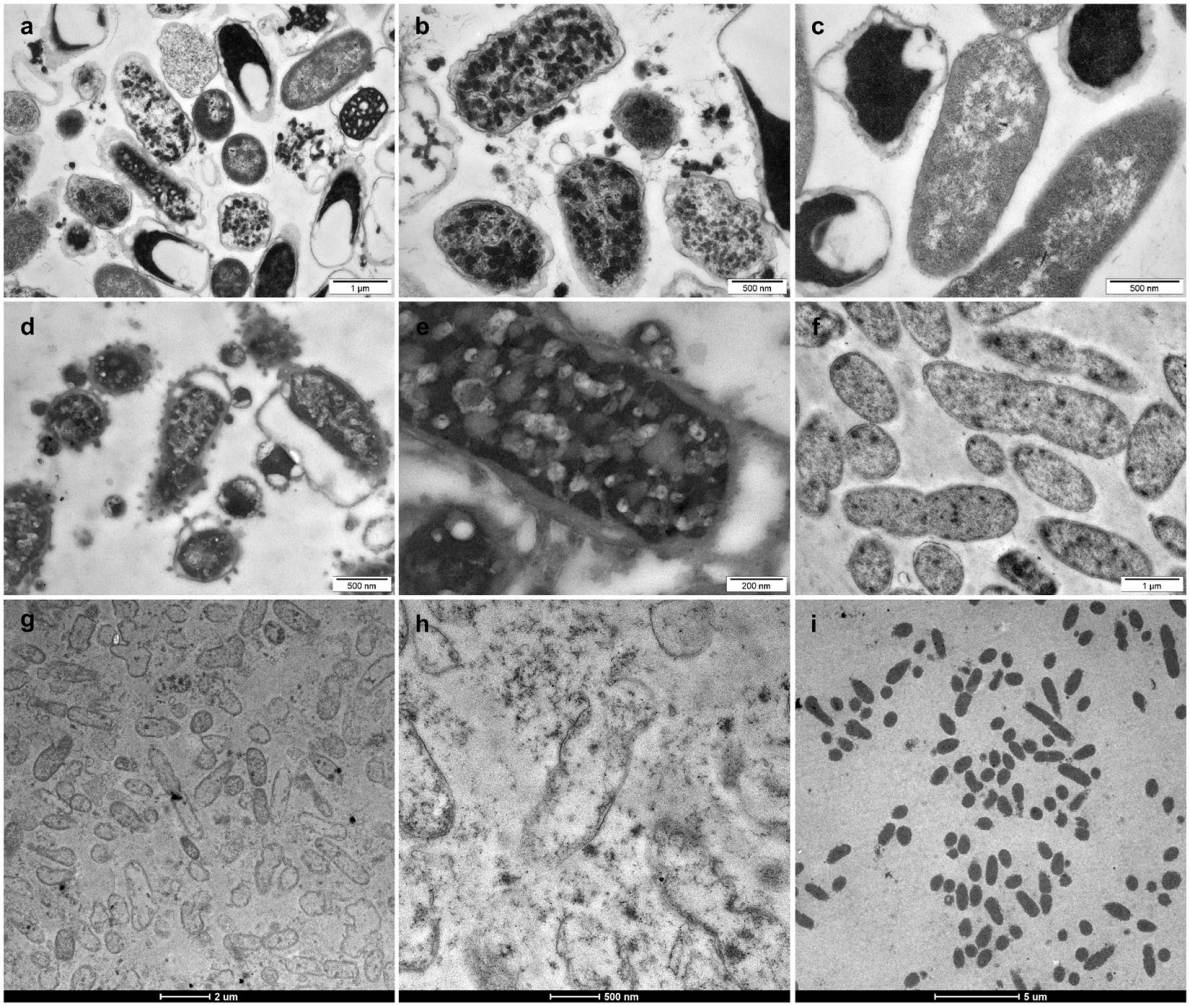
TEM analyses of HRNR-treated *E*. *coli* and *P*. *aeruginosa*. (**a**,**b**) Transmission electron microscopy (TEM) analyses of 6.25 × 10^7^/ml *E*. *coli* ATCC 11775 in 10 mM NaP, pH 5.5, 1 h treatment with 312.5 µg/ml rHRNR_2591–2684_. (**c**) *E*. *coli* control. Note the absence of membrane perturbation and the presence of intracytoplasmic electron-dense aggregates in rHRNR_2591–2684_-treated bacteria (**a**,**b**). The hyperhydrated looking periplasmic space of many cells in the control (a), is similar as seen for *E*. *coli* treated with low ion strength and acidic buffers^7^. TEM of 6.25 × 10^7^/ml *P*. *aeruginosa* ATCC 10145, in 10 mM NaP, pH 5.5, 1 h treatment with 312.5 µg/ml rHRNR_2591–2684_ (**d**,**e**). (**f**) *P*. *aeruginosa* control. Note condensation of electron-dense cytoplasmic material and blebs of the outer membrane with an occasional ballooning (**d**,**e**). 1 h treatment of 6.25 × 10^7^/ml *P*. *aeruginosa* ATCC 10145 with 469 µg/ml rSUMO3-HRNR_2591–2684_ in 10 mM NaP, pH 5.5 revealed widespread peeling of the outer membrane (**g**,**h**). (**i**) *P*. *aeruginosa* control. Images are representative of two independent experiments, sampling on average 10 images per condition and species in each experiment.

Treatment of *E*. *coli* ATCC 11775 with rHRNR_2591–2684_ for 2 h caused condensation of electron-dense cytoplasmic material, forming large aggregates, and in some cells apparent cytological lysis with liberation of electron dense material upon treatment at pH 5.5, in 10 mM Na- phosphate (NaP) (Fig. 1a). Incubation of control *E*. *coli* ATCC 11775 cells at pH 5.5, in 10 mM NaP, revealed in some bacteria an increased electron density of the cytosol. Here the periplasmic space of many cells looked hyperhydrated, very similar as previously reported^7^, but the inner and outer membranes remained intact (Fig. 1b). When *P*. *aeruginosa* ATCC 10145 was exposed to rHRNR_2591–2684_ at identical conditions, condensation of electron-dense cytoplasmic material and blebs of the outer membrane with an occasional blebbing were seen (Fig. 1c,d), showing evidence of inner membrane breakage. In the controls, *P*. *aeruginosa* cells did not show the signs of hyperhydration (Fig. 1e,h) seen in *E*. *coli* cells (Fig. 1b). Exposure of *P*. *aeruginosa* to rSUMO3-HRNR_2591–2684_ resulted in widespread peeling of the outer membrane. Many cells underwent extensive lysis and, as a result, lost most of the cytoplasmic electron-dense material. Nearly all cells exposed to this CIDAMP became ghost cells with complete extraction of cytoplasmic contents (Fig. 1f,g). Short HRNR-peptides like the duodecapeptide HRNR_1132–1143_ (GSGSRQSPSYGR) - the only *P*. *aeruginosa*-cidal CIDAMP with a positive net charge of +2 - and those of distinct HRNR-repeat domains like HR1-11 (HRNR_2606–2628_), HR2-11 (HRNR_1132–1157_) and HR1-18 (HRNR_2656–2677_), caused morphological changes (Supplementary Figs S1 and S2) very similar as seen for the long HRNR fragments (Fig. 1a,c,d). We then studied the kinetics of CIDAMP-dependent ultrastructural changes. Exposure of *E*. *coli* ATCC 11775 to rSUMO3-HRNR_2591–2684_ for 5 min caused a few blebs of the outer membrane, which was more prominent after 20 min exposure, showing morphological evidence of a bacterial stress response. Leakage with liberation of electron dense cytoplasmic material was not observed (Supplementary Fig. S3). Interestingly, at higher magnification nanofiber-like structures upon rSUMO3-HRNR_2591–2684_-treatment of *E*. *coli* were seen (Supplementary Fig. S3b). Since disordered proteins are prone to form nanofibrils and amyloid-like structures^8^ we surmised that rSUMO3-HRNR_2591–2684_ may form nanofibers. To test this, rSUMO3-HRNR_2591–2684_ was treated with ultrasound and then analyzed for amyloid-formation, confirming our hypothesis (Supplementary Fig. S4). Thus, nanostructures seen in samples of rSUMO3-HRNR_2591–2684_-treated *E*. *coli* (Fig. 1a and Supplementary Fig. S4) might have originated from rSUMO3-HRNR_2591–2684_, an observation supported by scaffolds of HRNR-nanofibers seen *in vivo* in the eye lid^2^.

**Figure 2:**
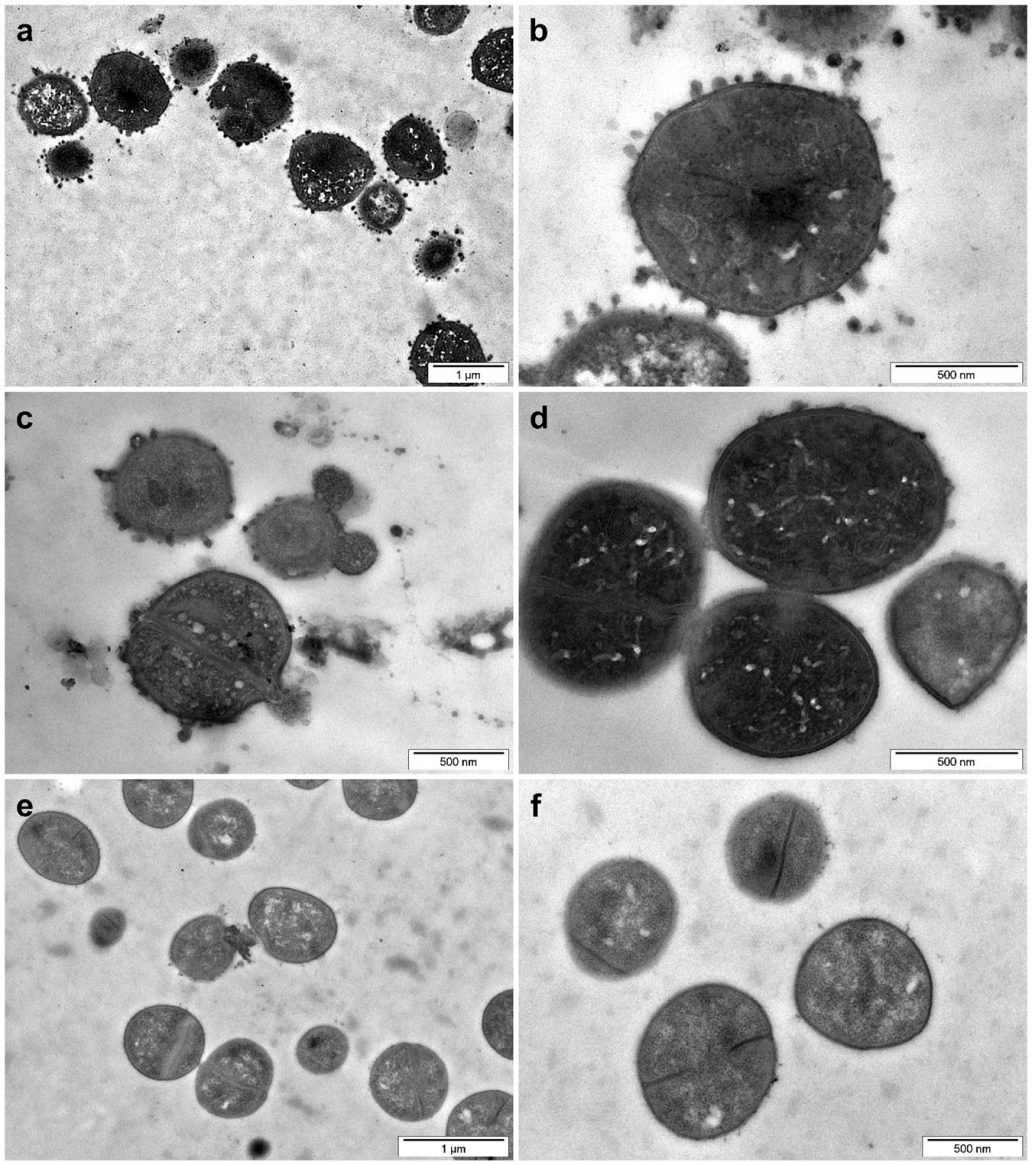
Ultrastructural analyses of rHRNR_2591–2684_-treated *S*. *aureus*. TEM analyses of 6.25 × 10^7^/ml *S*. *aureus* ATCC 6538, in 10 mM NaP, pH 5.5, treated with 312.5 µg/ml rHRNR_2591–2684_ for 2 h. Note the condensation of electron-dense cytoplasmic material and formation of membrane blebs (**a**–**c**). Occasionally ballooning (**c**) and aggregated cells (**d**), connected via electron-dense contacts, were found upon rHRNR_2591–2684_-treatment. (**e**,**f**) Control. Images are representative of two independent experiments, sampling on average 10 images.

Exposure of *S*. *aureus* towards HRNR_2591–2684_, at pH 5.5, elicited small blebs and - as seen in *P*. *aeruginosa* and *E*. *coli* - condensation of electron dense cytoplasmic material (Fig. 2). HRNR_2591–2684_ caused *S*. *aureus* aggregation, similar as observed in *E*. *coli* (Supplementary Fig. S3). Aggregated cells are connected via electron-dense contacts (Fig. 2d) - resembling features seen for the sweet water polyp *Hydra vulgaris*-derived antimicrobial peptide hydramacin-1 upon exposure towards *E*. *coli*^9^.

**Figure 3:**
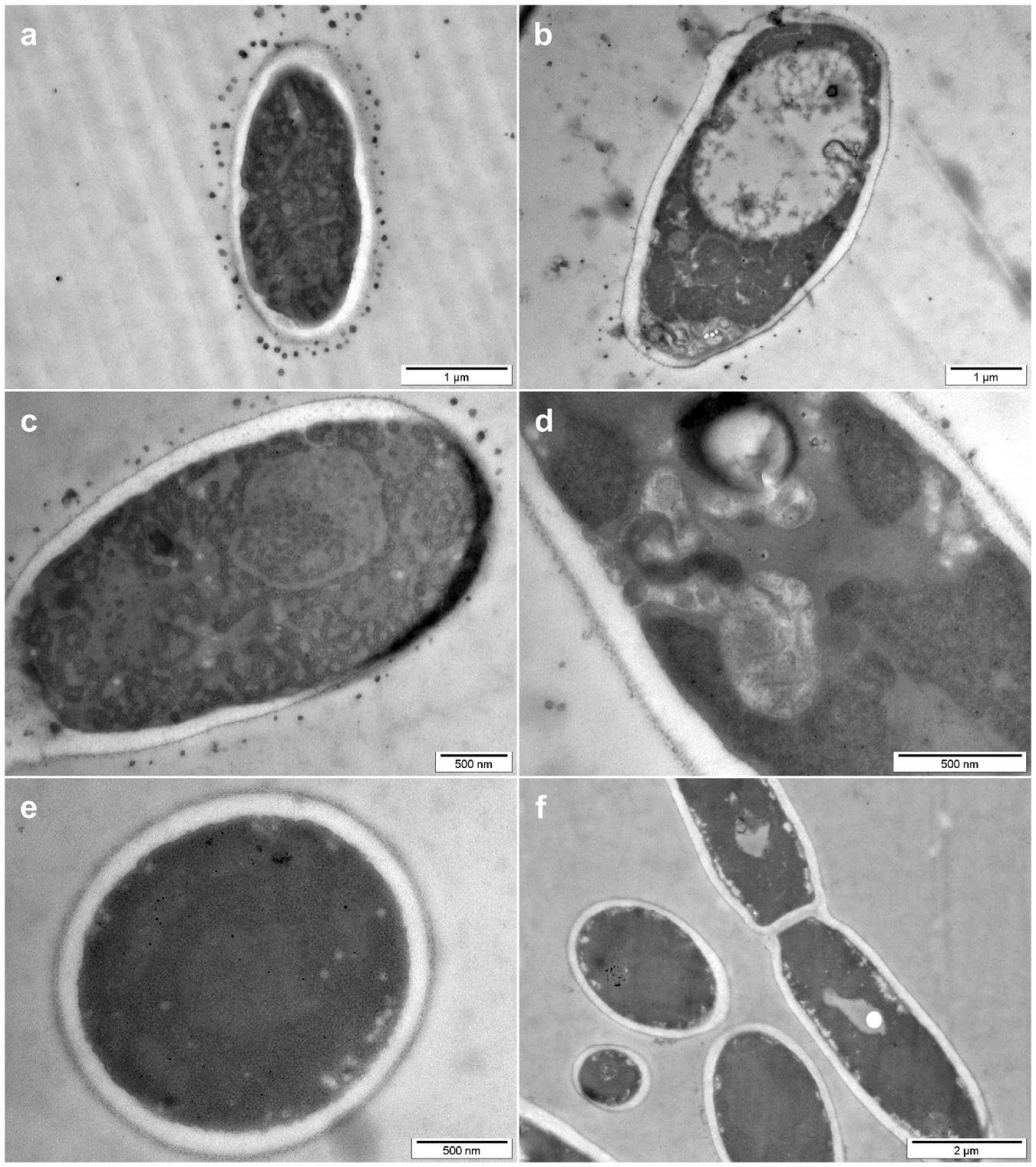
Ultrastructural analyses of HRNR-treated *C*. *albicans*. TEM analyses of 6.25 × 10^7^/ml *C*. *albicans* ATCC 244433, treated for 2 h with 312.5 µg/ml rHRNR_2591–2684_ in 10 mM NaP, pH 5.5 (**a**–**d**). (**e**,**f**) Control. Note the release of electron dense vesicles (**a**–**c**) and marked changes of the intracytoplasmic morphology. Images are representative of two independent experiments, sampling on average 10 images in each experiment.

Also treatment of the yeast *C*. *albicans* with rHRNR_2591_HRNR_2591–2684_ led to characteristic ultrastructural patterns with the release of electron-dense membrane vesicles and changes mainly of the nucleus, cytoplasmic structures and condensation and alteration of the chromatin (Fig. 3). Chromatin margination and condensation along the nucleus and blebs from the nucleus are hallmark ultrastructural signs of apoptosis in fungi^10^, indicating that rHRNR_2591–2684_ might kill *C*. *albicans* similar as AMPs like lactoferrin, human ß-defensins or plant defensins by apoptosis-like cell death^11^.

In summary, ultrastructural analyses of rHRNR_2591–2684_-treated bacteria like *P*. *aeruginosa*, *E*. *coli* and *S*. *aureus* reveal for various different HRNR-fragments no signs of immediate membrane alteration – typical features of pore-forming amphipathic antimicrobial peptides^5^. Instead a unique cytosolic aggregation of electron-dense particles, indicative for protein misfolding or assembly of misfolded polypeptides into cytotoxic aggregates^12^, was observed. In contrast, rHRNR_2591–2684_ causes in *C*. *albicans* ultrastructural changes reminiscent to fungal apoptosis-like programmed cell death.

## The Uptake of HRNR-Derived CIDAMPs is an Active Process

Although the action principle of the majority of yet known amphipathic AMPs is based on membrane permeabilization^5,13^, there is ample evidence now that some, or even most of these AMPs affect microbial viability also by other mechanisms, in addition or alternative to their membrane-permeabilizing/disrupting properties^5,14^.

The main characteristics of amphipathic AMPs for high binding and selectivity toward microbial membranes are the amino acid composition and sequence^15^. These determine the physicochemical properties of the peptide in respect to charge, amphipathicity, hydrophobicity, flexibility and H-bonding capacity as key factors for their mode of action and selectivity toward microbial cells^16^. The antimicrobial mechanism of classical amphipathic and cationic AMPs mostly relates to targeting the microbial cytoplasmic membrane by creating transmembrane pores or channels that cause leakage of intracellular molecules, which finally leads to cell death^17,18^. For AMPs having intracellular targets, the precise mechanism how bacterial cells are entered is not clear. Due to their small size, AMPs seem to diffuse rapidly inside and outside of the cell membrane^19^.

**Figure 4:**
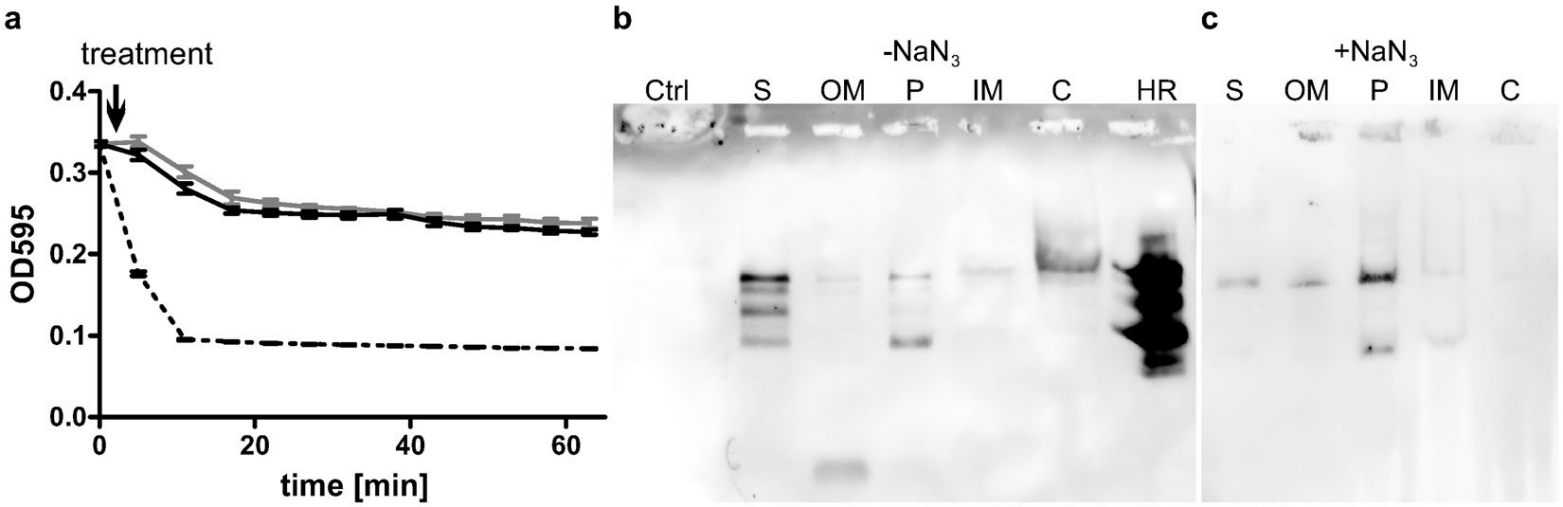
rHRNR_2591–2684_ is a non-permeabilizing, energy-dependently translocating CIDAMP. (**a**) rHRNR_2591–2684_ does not permeabilize the bacterial membrane. Lysis by 50 μg/mL lysozyme (expressed as OD_595_ against time ± s. e. m., n = 3) of chloramphenicol-treated *P*. *aeruginosa* PAO1 cells (gray line) in the presence of polymyxin B (PMB, 10 μg/mL; dotted line) or rHRNR_2591–2684_ (5 μg/mL; black line). (**b**) rHRNR_2591–2684_ is translocated into bacterial cytosol. HRNR-Western blot of fractionated, rhHRNR_2591–2684_-treated PAO1, Ctrl: control (untreated PAO1). S: sample supernatant, OM: outer membrane-, P: periplasmic-, IM: inner membrane-, C: cytoplasmic fraction, HR: Hornerin fragment rHRNR_2591–2684_. (**c**) rHRNR_2591–2684_ translocation is energy-dependent. PAO1 was treated with rHRNR_2591–2684_ in the presence of NaN_3_, fractionated and analyzed by HRNR-Western blot.

To get insight into the mechanism how HRNR-based CIDAMPs may traverse bacterial membranes, we analyzed the permeabilizing properties of rHRNR_2591–2684_ and its partition in subcellular compartments in *P*. *aeruginosa* PAO1. Confirming our TEM data, no membrane permeabilizing properties of this CIDAMP were found (Fig. 4a). Using fractionation techniques, we detected immunoreactive rHRNR_2591–2684_ in treated bacteria within the cytosol (Fig. 4b) upon Western blot analyses. Interestingly, pretreatment of the bacteria with the respiratory chain blocker sodium azide caused accumulation of rHRNR_2591–2684_ mainly in the outer membrane and periplasmic fraction (Fig. 4c) - indications for a passive interaction of this CIDAMP with the bacterial envelope and an active uptake mechanism of rHRNR_2591–2684_ into bacterial cytosol. Intriguingly, this observation appears to be similar to the uptake of certain colicin bacteriocins into *E*. *coli*. Here, an intrinsically disordered N-terminal domain facilitates the translocation across the outer membrane and is involved in an energy-dependent, TonB-mediated uptake^20^.

There are three proposed mechanisms by which cationic AMPs enter the cytoplasm of microbes: A spontaneous lipid-assisted translocation, a stereospecific receptor-mediated membrane translocation^21^ and a spontaneous translocation without pore formation. The main mechanism is most likely a spontaneous translocation with pore formation, as contemplated by the Shai-Matsuzaki-Huang model^13^. A second mechanism, based on translocation of the inner membrane peptide transporter SbmA, has been reported for several Pro-rich AMPs^22,23^. Uptake of the Pro-rich cathelicidin Bac7, which has an extended secondary structure^14^, occurs in a stereospecificity-dependent manner since the all-D-enantiomers were inactive as antimicrobials and were excluded from uptake into bacterial cells^24^. Since all-(D)-CIDAMPs we had investigated are similarly active as its all-(L)-enantiomers (TL, UG, Zhihong Wu, JB, AB, AT and JMS, Sci. Rep., in revision), a stereospecific cellular uptake of CIDAMPs seems to be less likely.

## HRNR-Derived CIDAMPs are Targeting Bacterial Ribosomal Proteins

**Figure 5:**
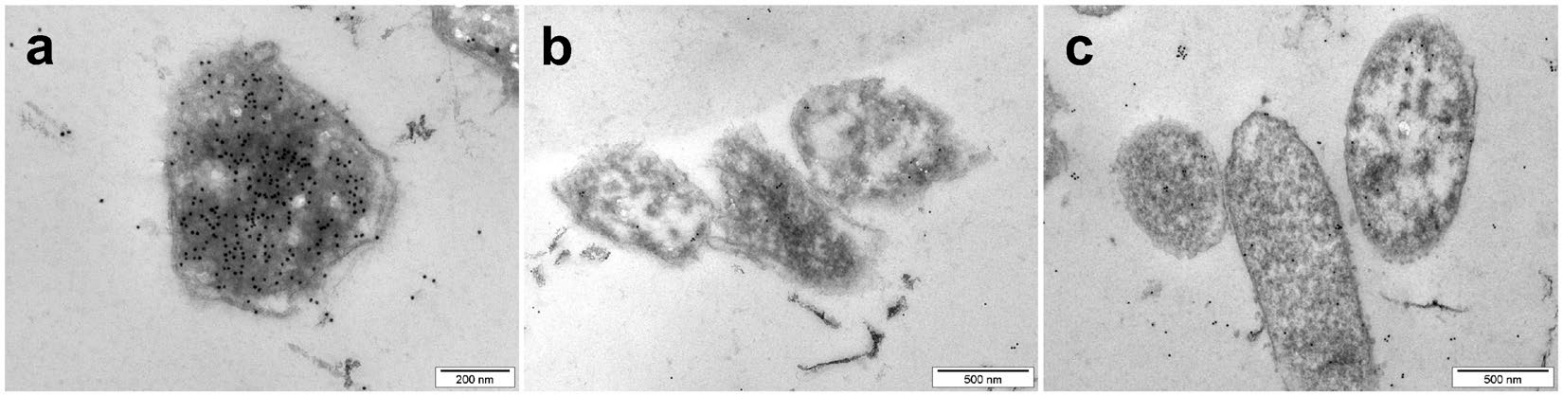
HRNR binds to intracytoplasmic aggregates in *P*. *aeruginosa*. *P*. *aeruginosa* ATCC 10145 was treated for 5 min with rHRNR_2591–2684_ (45 µg/ml 10 mM NaP, pH 5.5), and then cellular localization of this CIDAMP was analyzed by immunocytochemistry with a HRNR_2591–2684_-specific polyclonal antibody, followed by incubation with a gold-conjugated secondary antibody (**a**). Bacteria, treated with rHRNR_2591–2684_ (**b**) or buffer (**c**), followed by incubation with the gold-conjugated secondary antibody, served as controls. Note accumulation of intracytoplasmic immuno-gold (**a**), which is corresponding to electron dense cytoplasmic aggregates seen upon TEM-analyses of CIDAMP-treated *P*. *aeruginosa* (Fig. 1c,d). Images are representative of two independent experiments, sampling on average 10 images in each experiment.

Ultrastructural investigations of distinct bacteria treated with different HRNR-based CIDAMPs revealed electron dense cytoplasmic aggregates as a common characteristic (Fig. 4), assuming a unique killing mechanism for HRNR-derived CIDAMPs. To identify the intracellular target of rHRNR_2591–2684_, we performed immunogold-electron microscopy with rHRNR_2591–2684_-treated *P*. *aeruginosa* ATCC 10145 using post-embedding immunogold-staining techniques with an affinity-purified, rHRNR_2591–2684_-directed polyclonal antibody^1^ to localize cellular CIDAMP-binding sites. Exposure of *P*. *aeruginosa* ATCC 10145 to rHRNR_2591–2684_ caused gold particle accumulation at electron-dense aggregates within the cytoplasm, but not at the membrane, nourishing the hypothesis that rHRNR_2591–2684_ possibly binds to bacterial ribosomes (Fig. 5) and contrasting to the membrane interactions of Pro-rich AMPs, where the peptides localize uniformly around the *E*. *coli* membrane^25^. To delineate the mechanisms of the antimicrobial activity of CIDAMPs further, and following the hypothesis that bacterial ribosomes are the target of CIDAMPs, *E*. *coli* ribosomes were separated by SulfoLink®– coupling resin-chromatography, a method allowing its efficient isolation from bacterial lysates without harsh conditions and is rapidly enough to limit degradation, resulting in highly active ribosomes^26^. To explore whether selected CIDAMPs bind to ribosomal proteins, we analyzed SulfoLink®–chromatography fractions by a Far-Western blot analysis^27^, where protein-coated nitrocellulose membranes were incubated with rSUMO3-HRNR_2591–2684_ as “bait” protein and HRNR_2591–2684_-antibodies for visualization of HRNR-ribosomal protein interaction. Far-Western blot analyses of SulfoLink®–column-bound *E*. *coli*-proteins revealed multiple proteins interacting with HRNR_2591–2684_ (Supplementary Fig. S5).

56 ribosomal protein subunits, which to a high extent are posttranslationally modified^28^, have been identified in *E*. *coli* K12^28^, and its biochemical separation is a technically challenging task. Direct ion exchange HPLC allows the purification of only nine proteins^29^. RP-HPLC, however, was successfully utilized for separation of *E*. *coli* 30S ribosomal proteins and was shown to yield much higher numbers and greater resolution of these proteins than other HPLC-methods^30^ do. Also 50S ribosomal proteins could be separated by RP-HPLC^31^. Therefore we used RP-HPLC methods to yield both, *E*. *coli* 30S- and 50S-ribosomal subunits. Due to a limited solubility of distinct ribosomal proteins in different organic solvents, it was found to be impossible to separate all 50S and 30S ribosomal subunits at a single RP-HPLC-column and a single organic solvent as eluent^31^. We identified two different RP-HPLC columns (Jupiter® C18 widepore RP-HPLC column and an Aeris® C18 widepore RP-HPLC column) and gradients of two different eluents (either acetonitrile (ACN) in aqueous 0.1% trifluoroacetic acid (TFA) or 2-propanol (Prp) in aqueous 0.1% TFA) to be useful in this study.

Aliquots of each HPLC-fraction were applied either to a nitrocellulose membrane for a modified Far-dot blot analysis or to an SDS-PAGE gel for a Far-Western blot analysis^27^. Here, protein-coated nitrocellulose membranes were incubated with rHRNR_2591–2684_, biotinylated rSUMO3-HRNR_2591–2684_, biotinylated rHRNR_2591–2684_, biotinylated HR1-18, or rHRNR_1075–1172_. Thereafter, *Strep*-*Tactin*® or polyclonal antibodies^1^ against HRNR_2591–2684_ and HRNR_1075–1172_, respectively, together with biotinylated secondary antibodies, were used for visualization of HRNR-ribosomal protein-interaction.

Results indicate a specific binding of the recombinant biotinylated HRNR-derived CIDAMP rHRNR_2591–2684_ or its SUMO3-fusion protein to a panel of ribosomal proteins. We identified the *E*. *coli* 50S ribosomal proteins L2, L18, L22, L28 as well as the *E*. *coli* 30S ribosomal proteins S11, S13, S18, S19 and S20 when proteins of HPLC fractions were sequenced (Table 1, Supplementary Figs S6–10). Using a more sensitive LC-MS/MS analyses of HRNR-interacting ribosomal proteins, we identified, apart from the *E*. *coli* 50S ribosomal proteins L2, L18, L22, L28 and the 30S ribosomal proteins S11, S18, S19 and S20, in addition the 30S ribosomal proteins S3, S4, S6, S10, S12 as well as the 50S proteins L13 and L14 in HRNR-active HPLC fractions (Table 1, Supplementary Table S1, Figs S10–S12). Thus, the investigated CIDAMPs, rHRNR_2591–2684_ and biotinylated rSUMO3-HRNR_2591–2684_, may bind to a panel of distinct ribosomal protein subunits of *E*. *coli* (Table 1), each having unique affinity to HRNR polypeptides. Since resolution of SDS-PAGE analyses and RP-HPLC is not high enough for a complete separation of all different subunits, including truncated and posttranslationally modified forms, further studies are necessary to identify all HRNR-binding *E*. *coli*-ribosomal proteins and its specific affinity to distinct HRNR peptides and possibly other CIDAMPs.

**Table 1:** *E*. *coli* ribosomal proteins interacting with Hornerin*.

30S ribosomal proteins: ***S3***, ***S4***, *S5*, S6, *S7*, S10, ***S11***, ***S12***, ***S13***, ***S18***, **S19**, **S20**, ***S21***
50S ribosomal proteins: *L1*, ***L2***, *L3*, L4, *L5*, *L6*, L9, ***L13***, ***L14***, *L16*, ***L18***, *L19*, *L21*, *L22*, L23, ***L28***, *L31*

*Ribosomal proteins known to be phosphorylated^62^ are in italic, and those found at apparent relative high abundance in RP-HPLC-fractions (estimated via total ion current estimation upon ESI-MS analyses) are shown in bold.

One of the most prominent Far-Western blot bands of HRNR-binding ribosomal proteins corresponded to a 37 kDa protein (Supplementary Figs S5 and S6). Sequence analyses revealed the *E*. *coli* ribosomal 50S protein L2, a 29,729 Da protein^28^ migrating upon SDS-PAGE like a 38 kDa protein^32^. Thus, the *E*. *coli* ribosomal 50S protein L2 may be a major target of the investigated HRNR fragments, its biotin-derivatives and SUMO3-fusion protein.

Another major HRNR-binding protein seems to be the *E*. *coli* ribosomal 50S protein L22, which gave a strong 15 kDa band (Supplementary Fig. S8). This protein eluted as principle ribosomal protein upon HPLC-analyses with ACN as eluent (Supplementary Fig. S8b), but did not elute with Prp (Supplementary Fig. S6) – confirming previous findings^31^. We also noticed that L22, a 12,226 Da protein, migrates upon SDS-PAGE like a 15 kDa protein (Supplementary Fig. S8b), confirming a previous study^33^. Thus, two of the HRNR-stained bands (37 kDa and 15 kDa) correspond to the *E*. *coli* 50S ribosomal proteins L2 and L22. Due to close similarities of ribosomal proteins in its physicochemical properties^28^ and unknown affinities toward HRNR polypeptide fragments of the other, yet uncharacterized HRNR-binding ribosomal proteins, its structural analysis deserves further detailed investigations.

## Distinct HRNR-Derived CIDAMPs Reveal Similar Ribosomal Protein Binding Patterns

**Figure 6:**
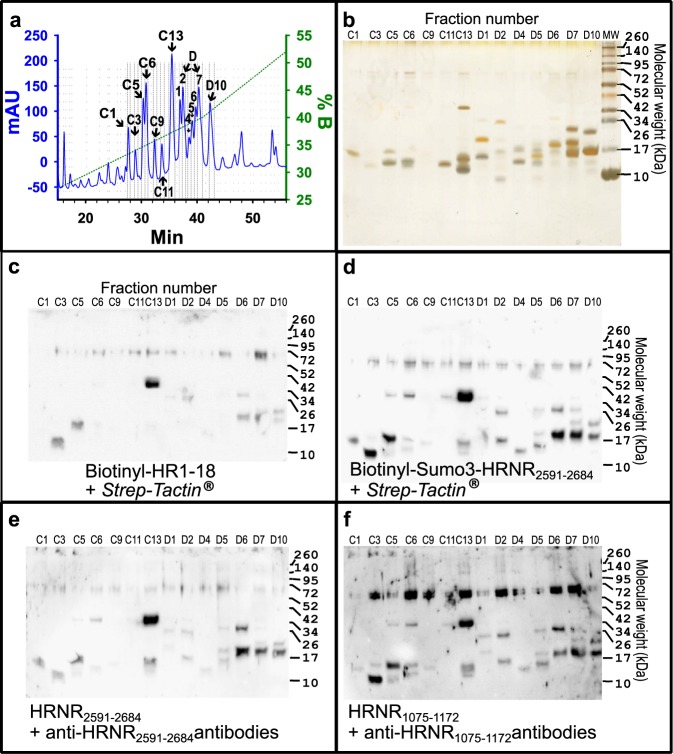
Distinct HRNR-derived CIDAMPs show similar ribosomal protein binding patterns. (**a**) SulfoLink®–column-bound proteins of an *E*. *coli*-extract were separated on a Jupiter® C18 RP-HPLC column with a Prp-gradient. HPLC fractions containing UV-absorbing peaks (C1–D10) were divided into five aliquots and adjusted in parallel to five PAGE-gels and separated. (**b**) Silver-stained proteins. (**c**) HRNR-Far-Western blot for probing with biotinylated HR1-18 (HRNR_2556–2677_) using *Strep*-*Tactin*®, (**d**) HRNR-Far-Western blot for probing with biotinylated rSumo3-HRNR_2591–2684_ using *Strep*-*Tactin*®, (**e**) HRNR-Far-Western blot for probing with rHRNR_2591–2684_ using anti-HRNR_2591–2684_ antibodies, (**f**) HRNR-Far-Western blot for probing with rHRNR_1075–1172_ using anti-HRNR_1075–1172_ antibodies. Note similarities of the staining patterns, irrespective the CIDAMP AA-sequence or biotin-labeling and irrespective whether a *Strep*-*Tactin*®- or antibody-detectable CIDAMP was used to probe and detect the target protein on the membrane. Note the presence of 70 kDa bands upon HRNR-Far-Western blot analyses in most of the investigated HPLC fractions with highest intensity for rHRNR_1075–1172_ binding (**f**). The most intensive band, corresponding to a 37 kDa protein in fraction number C13, was identified as *E*. *coli* ribosomal protein L2.

Next, we asked whether the structure of the CIDAMP and/or the method to detect CIDAMP-binding to bacterial ribosomal protein sub-units defines the outcome. We compared the binding patterns of four structurally different, HRNR-based CIDAMPs: biotinylated rSUMO3-HRNR_2591–2684_, biotinylated HR1-18 (HRNR_2656–2677_, which represents a 22-mer fragment of HRNR_2591–2684_), rHRNR_2591–2684_ and rHRNR_1075–1172_, each detected using the HRNR-Far-Western blot technique. Either *Strep*-*Tactin*® or antibodies against rHRNR_2591–2684_ and rHRNR_1075–1172_, respectively, were utilized (Fig. 6): SulfoLink®–column-bound proteins of an *E*. *coli*-extract were separated on a Jupiter® C18 widepore RP-HPLC column with a gradient of 2-propanol in aqueous 0.1% TFA and aliquots of fractions containing UV-absorbing peaks (C1–D10) (Fig. 6a) were adjusted in parallel to five PAGE-gels and analyzed for silver-stained proteins (Fig. 6b), for binding of biotinylated HR1-18 (Fig. 6c), biotinylated rSUMO3-HRNR_2591–2684_ (Fig. 6d), rHRNR_2591–2684_ (Fig. 6e), and for binding of rHRNR_1075–1172_ (Fig. 6f). We observed marked similarities of the staining patterns, irrespective whether a biotin-labeled or an antibody-detectable CIDAMP was used to probe and detect the target protein on the membrane. Biotinylated HR1-18 showed far less intensive bands, but an almost identical pattern with some bands missing (Fig. 6), which were weakly stained in experiments with long HRNR-peptides (Fig. 6). It is tempting to speculate that the affinity of the biotinylated 22-mer peptide HR1-18 towards distinct ribosomal proteins may be lower than that of the long HRNR-fragments. Most likely not only the peptide chain length, but also biotinylation of the CIDAMP affects its affinity to ribosomal proteins, a hypothesis supported by a comparatively much lower antimicrobial potency and efficacy of the biotinylated HRNR-peptide-fragments Biotin-rHRNR_2591–2684_ and rHRNR_2591–2684_ (TL, UG, Zhihong Wu, JB, AB, AT and JMS, Sci. Rep., in revision).

## Discussion

Our findings clearly show that distinct HRNR-derived CIDAMPs exert bactericidal activity in *E*. *coli* by targeting the ribosome. HRNR-Far-Western blot analyses identified several distinct HRNR-binding ribosomal proteins (Table 1 and Supplementary Figs S6–S12), among them the 50S ribosomal proteins L2, L22 and L28 as well as yet, for technical reasons, not be clearly assigned 14 additional 50S and 13 30S ribosomal subunits. Thus, distinct HRNR-derived CIDAMPs simultaneously target different sites of the bacterial protein synthesis machinery, suggesting a common mechanism of action.

The most intensive 37 kDa band seen upon HRNR-Far-Western blot analyses (Fig. 6) originated from *E*. *coli* 50S ribosomal protein L2. L2 is the second largest 50S ribosomal protein in *E*. *coli* and is one of the most highly conserved ribosomal proteins^34^ with numerous functions in protein synthesis^35,36^. It is absolutely required for subunit association as a primary rRNA binding protein and important for peptidyl transferase activity^36^. Thus, HRNR-derived CIDAMPs may interfere with these essential cellular processes.

The second clearly identified HRNR-interacting ribosomal protein is the *E*. *coli* 50S ribosomal protein L22, which is a component of the binding site for erythromycin on the ribosome^33^. L22 is important during the early stages of 50S assembly. It is one of the proteins that surrounds the polypeptide exit tunnel, where it can interact with nascent translation products in the exit tunnel, providing there one of the earliest contacts with a nascent peptide chain past the peptidyl transferase center^37^.

The third clearly identified HRNR-interacting protein is the *E*. *coli* 50S ribosomal protein L28 (Supplementary Fig. S7a,b), which is required for ribosome assembly^38^.

Therefore we surmise that CIDAMPs, by manifesting the features of several known classes of ribosome inhibiting antibiotics by simultaneously blocking the ribosome assembly, the peptidyl transferase center and the peptide-exit tunnel of the ribosome, and by targeting simultaneously multiple other ribosomal proteins, may cause the synthesis of aberrant and toxic proteins forming large disordered aggregates^12^ in CIDAMP-treated bacteria (Fig. 1 and Supplementary Figs S1–S3).

CIDAMPs show marked similarities to proline-rich antimicrobial peptides (PrAMPs) found in insects and some mammals. Like most CIDAMPs (TL, UG, Zhihong Wu, JB, AB, AT and JMS, Sci. Rep., in revision), PrAMPs are predominantly active against many Gram-negative bacteria^39^. Both, insect-derived PrAMPs and mammalian PrAMPs kill bacteria in a non-lytic mode of action by inhibiting bacterial protein translation at the 70S ribosome^40^. Crystal structure analyses of the PrAMPs revealed binding to the *Thermus thermophilus* 70S ribosome^41^. Each of the PrAMPs blocks the peptide exit tunnel of the ribosome by simultaneously occupying three well characterized antibiotic-binding sites and interferes with the initiation step of translation, thereby revealing a common mechanism of action used by these PrAMPs to inactivate protein synthesis^41^.

**Figure 7:**
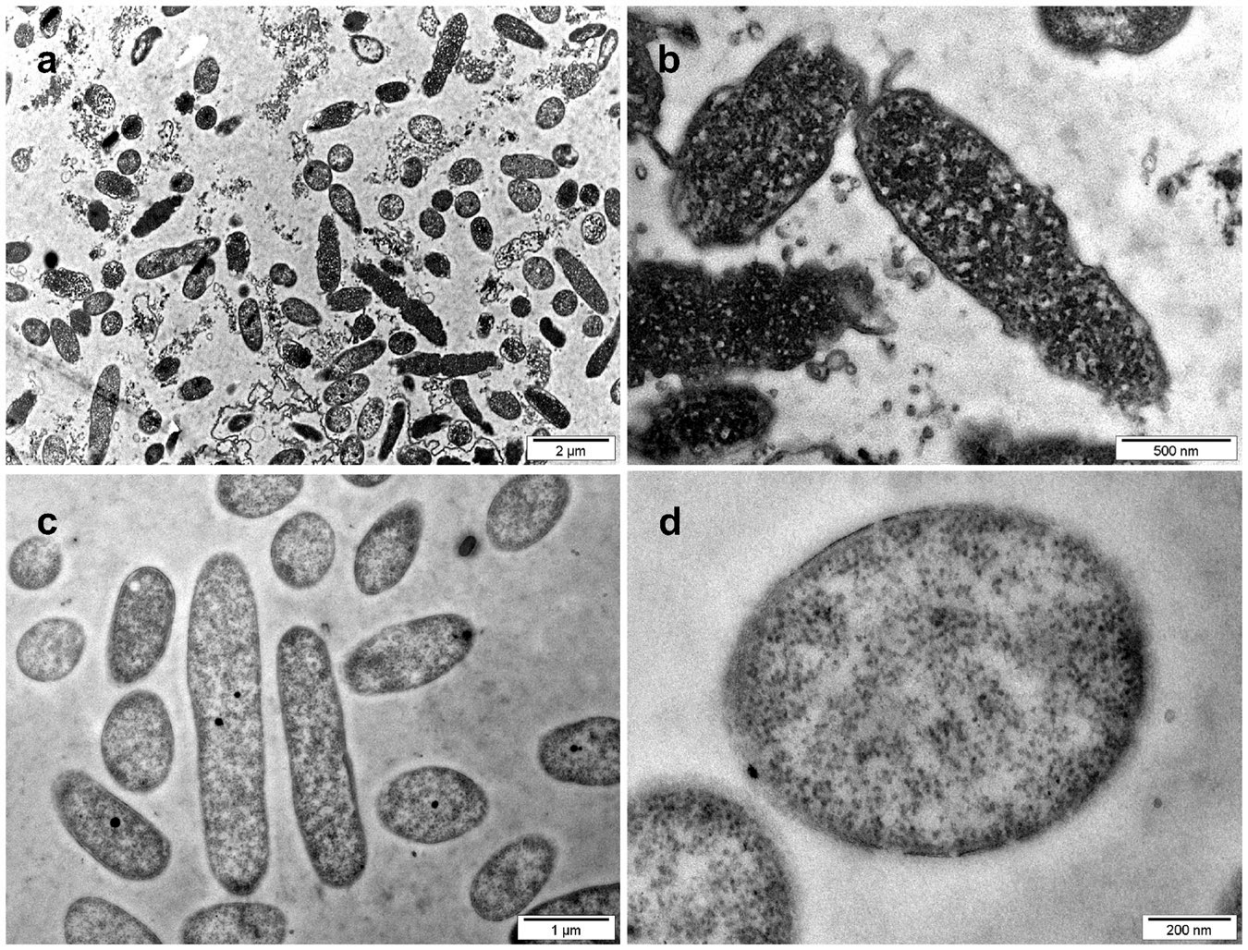
TEM of Gentamicin-treated *P*. *aeruginosa* at pH 5.5. (**a**,**b**) TEM of *P*. *aeruginosa* ATCC 11145, treated with 30 µg/mL Gentamicin in 10 mM NaP, pH 5.5, for 2 h at 37 °C. (**c**,**d**) Buffer control (also Supplementary Fig. S1g,h). Note condensation of electron-dense cytoplasmic material in Gentamicin-treated bacteria (**a**,**b**). Images are representative of two independent experiments, sampling on average 10 images.

The key role of the bacterial ribosome makes it an important target for antimicrobial agents and it is not surprising that a large number of clinically relevant antibiotics target this protein synthesis machinery of bacteria. The majority of antibiotics bind to one of three key sites in the ribosome: the decoding site (or A-site) on the 30S ribosome, the peptidyl-transferase center (PTC) on the 50S ribosome, and the peptide exit tunnel on the 50S ribosome^42^. Whereas aminoglycosides bind to the A-site and interfere with codon recognition and specificity causing synthesis of aberrant proteins, antibiotics such as chloramphenicol, clindamycin, and the oxazolidinone linezolid bind at the PTC and inhibit peptide bond formation. Macrolides such as erythromycin block elongation of the growing peptide chain at the peptide exit tunnel^43^. In support with our findings, ultrastructural analyses of aminoglycoside-treated *P*. *aeruginosa* shows marked electron-dense cytosolic aggregates (Fig. 7), very similar as seen upon challenge with almost all CIDAMPs we had studied (Fig. 1 and Supplementary Figs S1–S3). Since these aggregates are indicative for protein misfolding or assembly of misfolded polypeptides into insoluble and cytotoxic aggregates, known to be able to induce the bacterial death^12^, it is tempting to speculate that CIDAMPs, at least in part, are killing microbes in a self-assembly nanostructure-dependent manner. Several short cationic peptides, conjugated with fatty acids, mediate their antimicrobial activity from the formation of nanostructures^44^. Thus, it might be possible that at least some CIDAMPs may also express their antimicrobial activity via nanofibrils. May be, this is the case for the nanofiber-forming, HRNR-based CIDAMP rSUMO3-HRNR_2591–2684_ (Supplementary Fig. S4), which resembles several amphiphilic peptides, where the molecular self-assembly affects antibacterial properties^45^.

HRNR-derived CIDAMPs are also targeting *C*. *albicans* (TL, UG, Zhihong Wu, JB, AB, AT and JMS, Sci. Rep., in revision; Fig. 1). The mode of fungicidal action of these CIDAMPs is currently speculative. Ultrastructural analyses of HRNR-treated *C*. *albicans* (Fig. 3) revealed morphological signs reminiscent of apoptosis-like cell death (ALD) in fungi^10^. If fungicidal activity of CIDAMPs should be based on ALD, one may hypothesize that at least some CIDAMPs, due to their amyloidogenic properties might cause homologous seeding of toxic CIDAMP-nanostructures or seeding of heterologous, toxic protein aggregates.

Amyloidogenic peptides are toxic to bacterial cells^46^, and it was even proposed that the Alzheimer β-peptide may itself be an overlooked AMP, for which *C*. *albicans* was identified as the most sensitive microbial target^47^. Further, introduction of positively charged amino acids into other amyloidogenic peptides lead to the identification of novel potent AMPs with broad spectrum antimicrobial activity^48^, which would support the hypothesis that supramolecular structures of CIDAMPs may also have microbicidal properties. Formation of intracellular protein aggregates induces oxidative stress, including production of free radicals^49^. This would result in damage to other cellular proteins and induction of apoptosis in eukaryotic cells^50^.

Although the precise chain of events that leads to CIDAMP-dependent cell death in bacteria and *C*. *albicans* remains to be elucidated, the ability of CIDAMPs to elicit antimicrobial activity via protein aggregate-formation in bacteria represents a rich and unexplored chemical space to be mined in search of novel therapeutic strategies to fight infectious diseases^12^.

## Methods

### Synthetic Peptides

Peptides were purchased as TFA-salts from Genecust Europe (Luxembourg). When necessary, peptides were further purified by RP-HPLC, adopting conditions successfully used for purification of antimicrobial peptides^51^. The identity of the peptides was confirmed by ESI-MS analyses and its purity was better than 95% as determined by RP-HPLC and mass spectrometry.

Peptides were stored as a stock at 3 mg/mL in 0.01% (v/v) aqueous acetic acid at −20 °C until further use and dilutions were always freshly prepared.

### Recombinant expression of hornerin polypeptide fragments

Two recombinant hornerin polypeptides (rHRNR_1075–1172_, rHRNR_2591–2684_) were expressed in different expression systems (Supplementary Table S1). First, we attempted to generate HRNR repeat-domain peptides using a thioredoxin-reductase-(His)_6_-HRNR fusion protein, which had to be cleaved by enterokinase to liberate the full length HRNR-peptide. To subclone into the expression vector pET-32a (Novagen, North Ryde, Australia), PCR with *Pfu* DNA polymerase (Promega, Mannhein, Germany) was performed under the following conditions: 45 s at 98 °C; 5 cycles (45 s at 98 °C; 45 s at 55 °C; 1 min at 72 °C); 25 cycles (45 s at 98 °C; 1 min at 72 °C). The inserts were cut with *Bgl* II and *Not* I, gel purified and inserted into the pET-32a vector that had been double-digested with *Bgl* II and *Not* I. Although the fusion protein could be generated, all of our attempts to generate full length HRNR peptides failed. In particular the rHRNR_2591–2684_-fusion protein was found to be extremely sensitive towards treatment with enterokinase resulting in excessive degradation. Using a different strategy, we could generate defined HRNR polypeptides from SUMO3-His-tag-fusion proteins. To subclone the rHRNR_1075–1172_ into the expression vector pET-SUMO (Invitrogen), PCR was performed with *Pfu* DNA polymerase for 30 cycles (45 s at 98 °C; 45 s at Tm-5 °C; 1 min at 72 °C). The inserts were gel purified and inserted into the linerazied pET-SUMO vector. To subclone the rHRNR_2591–2684_ into the expression vector pSumo3 (LifeSensors, Malvern, USA), PCR was performed with *Pfu*Turbo Hotstart Polymerase (Stratagene/Agilent, Waldbronn, Germany) under the following conditions: 1 min at 98 °C; 10 cycles (30 s at 95 °C; 30 s at 53 °C; 1 min at 72 °C); 23 cycles (30 s at 95 °C; 30 s at 64 °C; 1 min at 72 °C). The Insert were cut with *Bsa* I and *Bam* HI, gel purified and inserted into the linearized pSumo3 vector. Specific primer pairs used in this study are listed in (Supplementary Table S2). All positive clones were identified and verified by sequencing. The plasmids were introduced into the *E*. *coli* host strain BL21(DE3)pLysS or Rosetta-gami 2(DE3)pLysS (Novagen). Subsequently, these were grown at 37 °C in TSB containing appropriate antibiotics. Expression of the recombinant protein was induced with 1 mM isopropyl thio-β-D-galactoside (IPTG) for 3 h at 37 °C. Bacteria were harvested by centrifugation at 5,000 × *g* for 5 min at 4 °C, lysed by sonication, centrifugated at 15,500 × *g* for 60 min at 4 °C (Beckman Coulter, Krefeld, Germany) and 0,45 µm filtered. Recombinant proteins were trapped with Ni^2+^ prepared columns (Macherey-Nagel, Dueren, Germany) and Ni^2+^-affinity column-bound proteins were subjected to preparative reversed-phase high-performance liquid chromatography (RP-HPLC) with a column (SP250/10 Nucleosil 300-7 C8; Macherey-Nagel) that was previously equilibrated with 0.1% (v/v) TFA in HPLC-grade water containing 10% acetonitrile. The polyhistidine-tagged fusion proteins were eluted with a gradient of increasing concentrations of acetonitrile containing 0.1% (v/v) TFA (at a flow rate of 3 mL/min). Fractions containing UV (215 nm)-absorbing material were collected, lyophilized and analysed by ESI-QTOF-mass spectrometry (Micromass, Manchester, U.K.). Purified histidine-tagged Sumo- and SUMO3-fusion proteins were then digested with SUMO protease 1 or 2 (Lifesensors) according to the manufacturer’s suggestion. The target polypeptide was purified by RP-HPLC on a Jupiter-5 µ-C4-300 Å HPLC column (Phenomenex, Aschaffenburg, Germany) equilibrated with 0.1% TFA in 10% acetonitrile. Peptides were eluted with a gradient of increasing concentrations of acetonitrile containing 0.1% (v/v) TFA (at a flow rate of 0.5 mL/min). Fractions of each peak were collected. Purity of recombinant fusion-proteins was determined by SDS-PAGE. Briefly, proteins were separated on NuPAGE^®^ Novex 10% Bis-Tris gels with MES SDS buffer (Invitrogen). Fusion proteins in Bis-Tris gels were stained with either silver nitrate (Sigma) or Coomassie blue R-250 (Sigma). The SeeBlue^®^ Plus 2 Pre-stained Standard marker (Invitrogen) was used as molecular weight markers. The polypeptide purity and molecular masses were assessed using QTOF-ESI-MS. Further details (in german language) are available from (http://macau.uni-kiel.de/receive/dissertation_diss_00018004).

### Biotin- and fluorescein-labeling of recombinant HRNR polypeptides

N-terminal biotinylation or fluorescein-labeling of recombinant HRNR polypeptides was performed with commercial biotinylation or fluorescein-labeling kits (Thermo Fisher Scientific^TM^) according to the manufacturer. The HPLC-purified biotinylated and fluorescein-labeled products were monitored by ESI-MS and were found to contain >90% of labeled polypeptides.

### Formation of rSUMO3-HRNR_2591–2684_ amyloid-like nanostructures

For the formation of amyloid-like nanostructures^52^, 1–2 µg lyophilized rSUMO3-HRNR_2591–2684_ or fluorescein-labeled rSUMO3-HRNR_2591–2684_ was dissolved in 100 µl distilled water. Samples were sonicated 5 times for 15 sec. at a power of 70% (Sonopuls GM70/Bandelin Elektronik) with intermissions of 15 sec, while cooling on ice. Samples were further analyzed for amyloid-formation using thioflavin T fluorescence monitoring^53^, for DAPI-staining and monitored by light microscopy (Supplementary Fig. S4).

### Microbes used in this study

*Candida albicans* ATCC 2443, *Escherichia coli* ATCC11775, *Pseudomonas aeruginosa* ATCC 10145, *Pseudomonas aeruginosa* ATCC 11446, *Pseudomonas aeruginosa* PA01, *Staphylococcus aureus* ATCC 6538.

### Microbial growth conditions

Bacteria were cultivated in either brain heart infusion medium (BHI), lysogeny broth (LB) or tryptic soy broth (TSB)^54^. If not otherwise stated, microbes were incubated under shaking conditions (37 °C at 170 rpm) or as recommended by ATCC or DSM. *Candida albicans* was cultured for 3 days on Yeast Extract-Peptone Dextrose (YPD) agar at 30 °C and yeast suspensions at appropriate density were treated with CIDAMPs as indicated.

### Colony-Forming Unit (CFU) assay of antimicrobial activity

All purified peptides and recombinant proteins were applied using a colony forming unit (CFU) assay in different media as described elsewhere (TL, UG, Zhihong Wu, JB, AB, AT and JMS, Sci. Rep., in revision).

### Transmission Electron Microscopy

All Transmission electron microscopy (TEM) imaging was performed by the Christian-Albrechts-University (CAU) Kiel Center of Biologic Imaging Core at the microscopy core facility. Logarithmic grown microorganisms were concentrated at ambient temperature to an OD_600nm_ of 4 in 10 mM NaP, pH 7.4/ 1% TSB or 10 mM NaP, pH 5.5/0.25% glucose, washed with the respective medium and then suspended to an OD_600nm_ of 2.0, depending on the species, corresponding to 10^9^–10^10^ microorganisms. The amount of the CIDAMPs used was about 2 × 10^7^ molecules per colony forming unit (CFU). 150 µL microorganism suspension (6.25 × 10^7^/mL) were then incubated with 10 µL CIDAMPs either at ambient temperature or at 37 °C for defined time periods (5 min, 30 min, 90 min, 120 min or 180 min) in the respective media (10 mM NaP, pH 5.5 with or without 0.25% (w/v) glucose or 1% TSB), which also served as controls to identify medium effects. Microorganisms were then fixed in 2.5% glutaraldehyde at 4 °C overnight. Bacteria were centrifuged at 3,220 × g for 10 min, supernatants discarded and then the pelleted samples were suspended at 44 °C (in a thermoblock) in a vial containing 2% Noble agar in distilled water. Samples in the vial were centrifuged, cooled to 4 °C and the bacteria-containing agar-block taken from the tip of the vial. This was then dehydrated in an ascending graded ethanol (EtOH) series. For embedding, the EtOH was replaced stepwise by a polyhydroxy-aromatic acrylic resin (LR White), starting at a resin:EtOH ratio of 1:2, followed by 1:1, 2:1, and three times in resin only, each for 30 min. Finally, samples were embedded in resin at 60 °C. The hardened resin was then cut into 5 nm sections and transferred onto a grid. All samples were analyzed with a transmission electron microscope (Philips TEM 208 or FEI Tecnai G2 Spirit BioTwin).

### Post-embedding immunogold electron microscopy

Logarithmic grown *P*. *aeruginosa* ATCC 10145 were concentrated at ambient temperature to an OD_600nm_ of 4 in 10 mM NaP, pH 5.5/ 0.25% glucose, washed with 10 mM NaP, pH 5.5/0.25% glucose and then suspended to an OD_600nm_ of 2. To 100 µL bacteria suspension, 10 µL rHRNR_2591–2684_ (500 µg/mL) or 10 mM NaP, pH 5.5/0.25% glucose for control were added and then incubated at ambient temperature for 5 min. Bacteria were fixed in 4% paraformaldehyde in 0.1 M NaP, pH 7.4, for 1 h, dehydrated and embedded in LR White resin. Ultrathin sections were prepared and mounted on nickel grids. After incubation with 1% BSA for 30 min, the sections were incubated with a HRNR_2591–2684_-specific polyclonal antibody (0.1 mg/ml in 0.1 M NaP, pH 7.4) at 4 °C overnight. After washing with 0.1 M NaP, pH 7.4, the sections were incubated with a rabbit anti-goat IgG, conjugated to 5 nm gold particles, (dilution 1:10) at ambient temperature for 3 h. The sections were then washed with water and stained with uranyl acetate. Transmission electron microscopy (TEM) imaging was performed by the Christian-Albrechts-University (CAU) Kiel Center of Biologic Imaging Core at the microscopy core facility with a Philips TEM 208 or FEI Tecnai G2 Spirit BioTwin.

### Studies on lytic properties of rSUMO3-HRNR_2591–2684_ in *P*. *aeruginosa*

*P*. *aeruginosa* ATCC11446 was grown over night at 37 °C and 170 rpm in LB medium without salt. 10 ml BHI medium was inoculated with an aliquot (1/50) of the overnight culture and incubated at 37 °C with shaking until an OD_600_ of 0.4. Bacteria were centrifuged (5 min 2,000 × g) and the pellet was washed once in 10 mM NaP, pH 7.4, and resuspended in 10 mM NaP, pH 7.4, supplemented with 15 µg/ml Chloramphenicol. The OD_600_ was adjusted to 0.8–1.0.

200 µl of the bacterial suspension was transferred into a round bottom microtiter plate. If appropriate, combinations of lysozyme (50 µg/ml) and Polymyxin B (1 mg/ml) or rSUMO3-HRNR_2591–2684_ (5 µg/ml) were added and bacterial lysis was followed by monitoring the OD_620_ over 1 h (measurements all 5 min) in a TECAN reader.

### Studies on the localization of rSUMO3-HRNR_2591–2684_ in bacterial compartments

2 ml of a *P*. *aeruginosa* PaO1 suspension was incubated at 37 °C/170 rpm overnight. A starter culture, prepared with 12 ml BHI/100 ml flask and 200 µl of an overnight culture, was incubated at 37 °C/170 rpm up to an OD_620_ of 0.4. The bacterial pellet was washed with 10 mM NaP, pH 5.5, containing 0.25% glucose, and re-suspended to an OD_620_ of 0.6 in the same buffer. If appropriate, 0.2% (w/v) NaN_3_, was added. Bacteria (100 µl) were treated with 100 µl of rSUMO3-HRNR_2591–2684_ (1 µg/ml) in 10 mM NaP, pH 5.5, containing 0.25% glucose or buffer (control) for 30 min at 37 °C statically. Thereafter bacteria were centrifuged (13,000 × g, 10 min, 4 °C) and supernatants were harvested. To isolate the periplasm, bacteria were resuspended in 100 µl 20% sucrose and 25 µl 5 mM EDTA, pH 8, followed by incubation for 10 min at 180 rpm and room temperature. The bacterial pellet was then spun down (13,000 × g for 10 min at 4 °C) and the supernatant removed. Thereafter, ice-cold water (100 µl) was added and the test mixture incubated for 10 min on ice at 180 rpm followed by 10 min storage on ice without shaking. Then the suspension was spun down (13,000 × g for 10 min at 4 °C) and the periplasm-containing supernatant was harvested.

To isolate the cytosolic content, the bacterial pellet was suspended in 100 µl ice-cold water, bacterial cells were broken up by heating (5 min at 95 °C) and, after centrifugation (13,000 × g for 10 min at 4 °C), the cytoplasm-enriched supernatant was harvested.

To isolate the inner membrane fraction, bacteria were washed once with 100 µl water. The bacterial cell pellet was suspended in 100 µl 1% Sarkosyl in 30 mM TRIS, pH 8, and incubated for 30 min at 37 °C under shaking conditions and subsequently spun down at 20,000 × g for 1 h at 4 °C. Whereas the inner membrane fraction was enriched in the supernatant, the remaining pellet contained the outer membrane fraction. The harvested bacterial compartments were separated by SDS-PAGE and rSUMO3-HRNR_2591–2684_ was detected by Western blotting.

### Ribosome isolation by SulfoLink^TM^ affinity chromatography

*E*. *coli* ATCC 11775 was inoculated into 10 mL TSB and incubated slantwise overnight and then cultured in 500 mL TSB at 37 °C/175 rpm until an OD_600_ (max.: 0.6–0.8) was reached. The culture was spun down at 13,000 × g for 10 min and the pellet was suspended in 10 mL 10 mM NaP, pH 7.3. After centrifugation (13,000 × g for 10 min, 4 °C), the sediment was re-suspended in 5 mL *E*. *coli* lysis buffer (20 mM Tris-HCl (pH 7.5), 10.5 mM Mg-acetate, 60 mM NH_4_Cl, 0.5 mM EDTA, and 3 mM β–mercaptoethanol), 5 × treated with ultrasound (70 sec, 15 sec., 30 sec. brake) and finally spun down at 20,000 × g for 30 min at 4 °C. The supernatant was sterile filtered and immediately applied to a SulfoLink^TM^-affinity column (Thermo Fisher Scientific^TM^). Bound material was eluted with elution buffer according to the protocol provided by the manufacturer. Eluted material (“crude ribosomes and ribosomal proteins”) were stored at 4 °C until further use.

### Generation of a crude ribosomal subunit-preparation for HPLC-separation

For isolation of ribosomal subunits, SulfoLink ^TM^- affinity column-enriched ribosomes were treated according to a protocol recently described^55^. Briefly, the affinity column eluate (2.7 mL) was supplemented with 300 µl 1 M MgCl_2_. Then 6 mL glacial acetic acid was added and the sample was stirred for 45 min on ice. Precipitated RNA was removed by centrifugation (10,000 × g for 10 min, 4 °C). Proteins were precipitated by adding 5 vol. ice-cold acetone to the supernatant and stored at −20 °C overnight. Unlike the original protocol, which recommended washing the sediment with cold acetone, we omitted this step due to dramatic losses of material and problems to precipitate proteins again. The pellet was dried in vacuum for 5 min., dissolved in 0.1% (v/v) TFA in water containing 4 M urea (37 °C, 1 h shaking).

### Analytical RP-HPLC of ribosomal proteins

Separation of *E*. *coli* ribosomal subunits was performed by reversed-phase (RP) high performance liquid chromatography (HPLC), using separation strategies successfully performed for separation and isolation of cationic antimicrobial peptides and chemokines^56–58^. Prior to injection into the HPLC-column, crude ribosomal subunit preparations in TFA-water/urea were spun down and the supernatant was separated by RP-HPLC. We either used a Jupiter® 300 Å, 250 × 12.6 mm, C18-RP-HPLC column (Phenomenex) or an Aeris® widepore XB C18, 250 × 12.6 mm, RP-HPLC column (Phenomenex) and either a gradient of acetonitrile (ACN) in aqueous 0.1% TFA, or a gradient of 2-propanol (Prp) in aqueous 0.1% TFA – as indicated for separation of ribosomal proteins. The presence of proteins was monitored at 215 nm, 254 nm and 280 nm. Fractions were collected according to the presence of UV (215 nm) absorbing peaks.

It is important to note that samples to be eluted either with an ACN- or Prp-gradient have been tested for possible precipitate formation by these solvents (80%) prior to application. Precipitate-depleted application samples were then lyophilized, dissolved in 0.1% TFA and then injected into the RP-HPLC column. Nevertheless, we often faced high pressure problems, possibly due to precipitation of some of the ribosomal subunits as a result of stationary phase interaction. This also caused an apparently more or less ribosomal subunit-selective depletion of at least some of the ribosomal subunits, which were missing in the eluates. The precipitation/binding of ribosomal subunits to the column material caused different UV-absorbance profiles of the eluates when repeating the HPLC-separation with additional crude ribosomal protein preparations.

### SDS-PAGE-analyses and protein staining

Electrophoretic mobility of ribosomal proteins was investigated using 12% SDS-polyacrylamide gels (SDS-PAGE) in the presence of 8 M urea and tricine^59^, as described for chemokines and antimicrobial peptides^51,60^. Peptides were visualized by silver staining or by Far-Western blot analysis (see below).

SDS-PAGE separation of selected RP-HPLC fractions was performed simultaneously with five different gels, where one gel was silver-stained and the other four were used for Far-Western blot-analysis. To achieve this, all HPLC-fractions were lyophilized within the vials and then the residues in each vial were dissolved in 55 µl 0.01% (v/v) acetic acid, followed by blending with 20 µl of 4 × SDS-PAGE loading buffer, which contained 1 mg/mL DTT. The mixtures, corresponding to the selected fractions, were heated to 95 °C for 5 min, centrifuged and then 14 µl of each were applied to the five different gels and separated.

### HRNR-Far-Western-blot-analyses

For HRNR-Far-Western-blot analysis, ribosomal proteins-containing samples (14 µl each, see above) were separated on a 12% SDS-tricine polyacrylamide gel containing 8 M urea. Proteins were then transferred to a nitrocellulose membrane (pore size: 0.2 µm, Schleicher & Schuell BioScience, Dassel, Germany) or polyvinylidenfluoride membrane (pore size: 0.2 µm, GE Healthcare) using an alkaline transfer buffer (48 mM Tris, 39 mM glycine, 0.0375% (w/v) SDS and 20% EtOH (pH 9.2)). An alkaline transfer buffer is essential to get cationic proteins efficiently transferred to a membrane^1^. Thereafter, the membrane was blocked for 1 h in blocking buffer (5% (w/v) bovine serum albumin (BSA) in PBS/Tris, pH 7.4 + 0.05% Tween) and, after washing with PBS/Tris, used for further Far-Western blot analyses: Membranes were incubated with biotinylated HRNR_2591–2684_ (4 µg/mL) in PBS/Tris, biotinylated HR1-18 in PBS/Tris (4 µg/mL), recombinant HRNR_2591–2684_ (4 µg/mL) and recombinant HRNR_1075–1172_ (4 µg/mL), respectively, at 4 °C overnight. This was followed by a 4 × wash with PBS/Tris, pH 7.4. Thereafter, membranes were incubated on a rotator overnight, with either Streptactin®-HRP (iba-lifesciences) in PBS/Tris, pH 7.4 (1:20,000) for 1 h (for biotinylated HRNR-peptides), or with goat-anti-HRNR_2591–2684_ (1 µg/mL)^1^ in PBS/Tris, pH 7.4, and with goat-anti-HRNR_1075–1172_ (1 µg/mL)^1^ in PBS/Tris, pH 7.4, for recombinant HRNR-peptides, respectively. Membranes were then washed 6-fold with PBS/Tris, and incubated with peroxidase substrate (Roche Lumilight Western Blotting Substrate No. 12015196001) in the case of biotinylated HRNR peptides. The other membranes were then incubated with mouse anti goat IgG-HRP (Jackson, No. 205-035-108) 1:10,000 at 4 °C overnight. After an additional washing step (6×) with PBS/Tris, pH 7.4, membranes were incubated with a peroxidase substrate (Roche Lumilight Western Blotting Substrate No. 12015196001) at ambient temperature and documented with a “Diana III Digital CCD Imaging System” or “FUSION FX7”.

### HRNR-Far-Immuno-Dot-blot-analysis

10–30 µL-aliquots of RP-18-HPLC-fractions were lyophilized in a microtiter plate, each residue dissolved in 5 µL of water, and 2 µL applied to a nitrocellulose membrane (0.2 µm-pore size; Bio-Rad) in a DotBlot System (Manifold I). After blotting, the membrane was saturated with PBS/M [PBS, pH 7.4, containing 5% (wt/vol) freeze-dried low-fat milk or BSA] at ambient temperature for 1 h and then washed three times with PBS/T [1 × PBS containing 0.05% (vol/vol) Tween 20]. Membranes were then incubated overnight with biotinylated rSUMO3-HRNR_2591–2684_ (4 µg/mL) in PBS/T or recombinant HRNR_2591–2684_ (4 µg/mL) in PBS/T at 4 °C. This was followed by a 4x wash with PBS/T. Thereafter, membranes were incubated on a rotator, either with Streptactin®-HRP (iba-lifesciences) in PBS/T (1:20,000) for 1 h to detect biotinylated rSUMO3-HRNR_2591–2684_, or overnight with goat-anti-HRNR_2591–2684_ (1 µg/mL)^1^ in PBS/T to detect rHRNR_2591–2684_. Membranes were then washed 6-times with PBS/T and incubated with peroxidase substrate (Roche Lumilight Western Blotting Substrate No. 12015196001) in the case of biotinylated HRNR peptides. Other membranes were then incubated overnight with mouse-anti-goat HRP 1:20,000 (Invitrogen-thermofisher) in PBS/T at 4 °C. After an additional 4x wash with PBS/T and twice with PBS, membranes were incubated with a peroxidase substrate (Roche LumiLight or SuperSignal West Dura; Pierce) at ambient temperature for 5 min and the chemiluminescence was monitored with a “Diana III Digital CCD Imaging System” (Raytest) or “FUSION FX7” (Filber). Biotinylated rSUMO3-HRNR_2591–2684_ (50 ng/dot) or rHRNR_2591–2684_ (50 ng/dot) served as positive controls.

### Amino acid sequencing of ribosomal proteins

For amino acid sequencing of proteins, RP-HPLC fractions were lyophilized, and the residues dissolved in 0.01% acetic acid (v/v), reduced with DTT, alkylated with iodoacetamide followed by digestion of the alkylated peptides with a mixture of sequencing grade LysC and modified trypsin as described^51^. Tryptic digests were then subjected to MS/MS-analyses on a QTOF-2 instrument utilizing electrospray ionization and data subjected to Mascot database search (http://www.matrixscience.com/)^61^. MS/MS sequence evaluation of peptide fragments was done using MassLynx PepSeq software.

### Tryptic digestion of HPLC-fractions for nano-LC-ESI-MS of ribosomal proteins

Samples have been filled up with ammonium bicarbonate buffer (50 mM) to a final volume of approximately 50 µL. To reduce disulfide linkages, 1 µL TCEP solution (100 mM) was added and the samples heated to 60 °C for 20 minutes. Cysteine residues were blocked through alkylation by adding 1 µL acrylamide solution (200 mM) and incubated at room temperature for 20 min. For digestion, 50 ng trypsin was added and incubated at 37 °C for 4 h. The digest was stopped by adding 1 µL TFA.

10 µL of the digested samples were injected into a Dionex U3000 nano-LC system (Dionex, Idstein, Germany) coupled online to a Q Exactive Orbitrap mass spectrometer (Thermo Fisher Scientific, Bremen, Germany). Peptides were desalted on a trap column (Acclaim Pepmap C-18, 300 µm × 5 mm, 5 µm, 100 Å, Dionex) at a flow-rate of 30 µL/min with loading buffer for 2 min before being eluted onto an analytical column (Acclaim Pepmap C-18, 75 µm × 500 mm, 3 µm, 100 Å, Dionex) at a flow-rate of 300 nL/min. For peptide elution and separation, a linear gradient with eluent A (0.05% FA) and eluent B (0.04% FA in 80% ACN) was employed: 4–50% B in 28 min, 50–90% B in 5 min, 90% B for 10 min, 90–4% B in 0.1 min and 4% B for 15 min. MS data were recorded from 5 to 60 min. MS full scans at a resolution of 70,000 were acquired between 300 and 2,000 m/z. The 10 most intense precursors with a charge state of at least 2+ and at most 7+ were isolated and fragmented using HCD and a normalized collision energy of 25% applied (isolation width was set to 3 m/z). The resolution for MS/MS acquisition was set to 17,500. After fragmentation, precursors were excluded from further isolation for 15 sec.

Data interpretation was performed using the Proteome Discoverer software (version 1.4, Thermo Fisher) and the search engine Mascot (version 2.2.07, Matrix Science, London, UK). All precursors between 350 and 5,000 Da with a signal-to-noise ratio of at least 1.5 were considered. The search was performed against a FASTA database of the combined human and E. coli proteomes (76377 sequences, source: www.uniprot.org, last updated in 04/2016) with tryptic enzyme specificity selected.

Precursor and fragment mass tolerances were set to 10 ppm and 0.02 Da, respectively. Oxidation of methionine, acetylation of N-termini and deamidation of asparagine and glutamine residues were set as dynamic modification. Propionamidation of cysteine residues was set as only static modification. All sequences were also searched against a decoy list of peptides with a strict (0.01) and relaxed (0.05) false discovery rate (FDR). For identification, only high confident peptide spectrum matches were allowed.

## Electronic supplementary material

Dataset 1
