# Supplementary material for: Hornerin contains a Linked Series of Ribosome-Targeting Peptide Antibiotics
Source: Sci Rep. 2018 Nov 1;8:16158. doi: 10.1038/s41598-018-34467-8 (PMC6212518; doi:10.1038/s41598-018-34467-8)
Supplement: Supplementary file 1 — Dataset 1 [file 41598_2018_34467_MOESM1_ESM.pdf]

Supplementary information

Hornerin contains a Linked Series of Ribosome-Targeting Peptide Antibiotics

Ulrich Gerstel[1,*], Ties Latendorf[1,*], Joachim Bartels[1], Alexander Becker[2], Andreas Tholey[2], Jens-Michael Schröder[1,§]

1 Dpt. of Dermatology, University-Hospital Schleswig-Holstein, Campus Kiel, Kiel, Germany

2 Institute for Experimental Medicine – AG Systematic Proteomics & Bioanalytics, Kiel University (CAU), Kiel, Germany

§ corresponding author

* equal contribution

Supplementary Figure S1

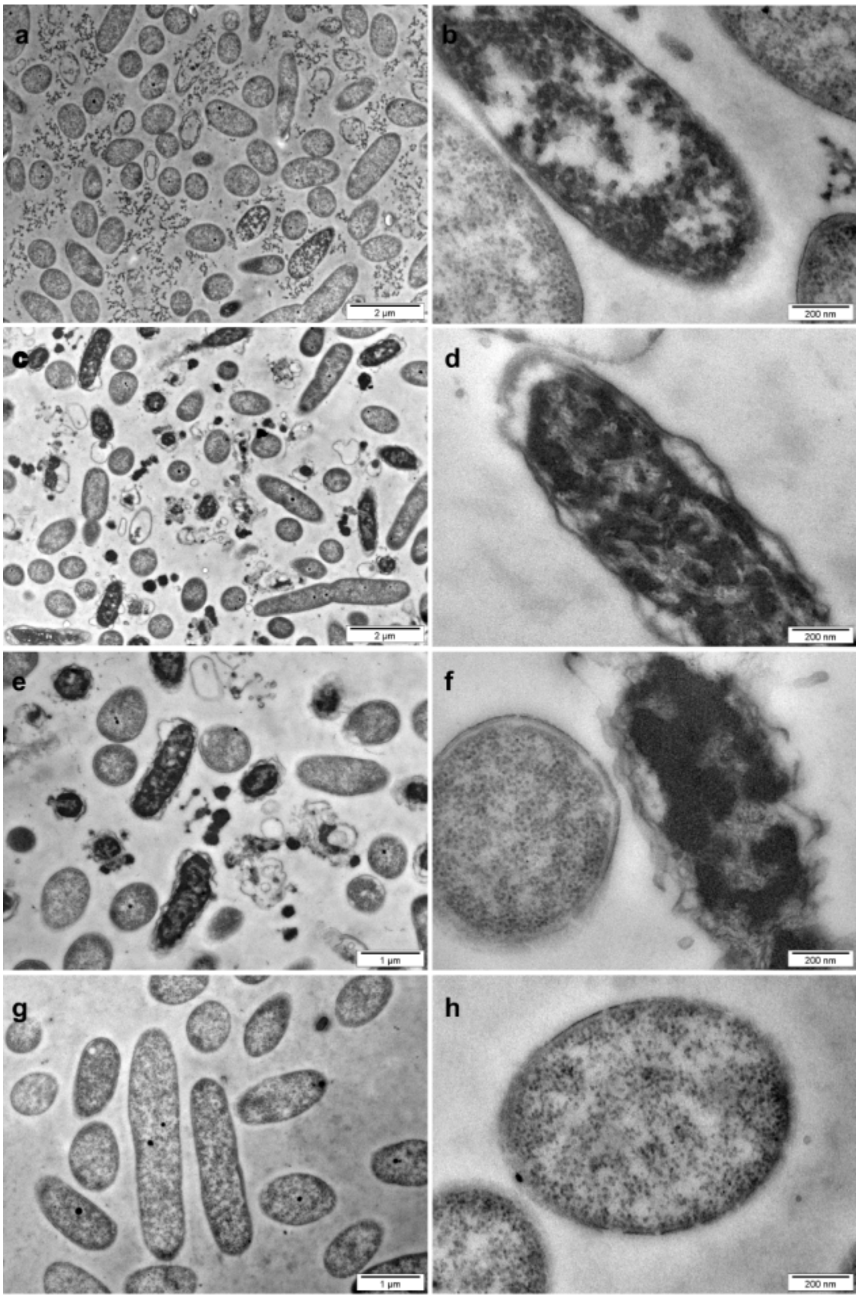

Supplementary Fig. S1 | TEM of *P. aeruginosa*, treated with selected HRNR-peptides.
TEM analyses of 6.25 x 10^7/ml *P. aeruginosa* ATCC 11446, in 10 mM NaP/ 1 % TSB/ pH 5.5, treated with 667 µg/mL HRNR$_{1132\text{-}1143}$ (GSGSRQSPSYGR) (**a, b**), 667 µg/mL HRNR$_{1132\text{-}1157}$ (GSGSRQSPSYGRHGSGSGRSSSSGQH) (**c, d**) and 667 µg/mL HRNR$_{2606\text{-}2628}$ (HGSRSGQSSRGERHGSSSGSSSH) (**e, f**), respectively, for 1h at room temperature. Note the dodecapeptide HRNR$_{1132\text{-}1143}$ affecting only a limited number of cells, which are destroyed (**a, b**) in a similar fashion as seen with rSUMO3-HRNR$_{2591\text{-}2684}$ with widespread peeling of the outer membrane and extensive lysis with loss of the cytoplasmic electron-dense material. A limited number of *P. aeruginosa* ATCC 11446, treated with either HRNR$_{1132\text{-}1157}$ (**c, d**) or HRNR$_{2606\text{-}2628}$ (**e, f**) showed large blebs on the outer membrane. Cytoplasmic swelling was observed at the area where the outer membrane appeared to be ruptured. **g, h**, Control. Images are representative of two independent experiments, sampling on average 10 images per condition in each experiment.

Supplementary Figure S2

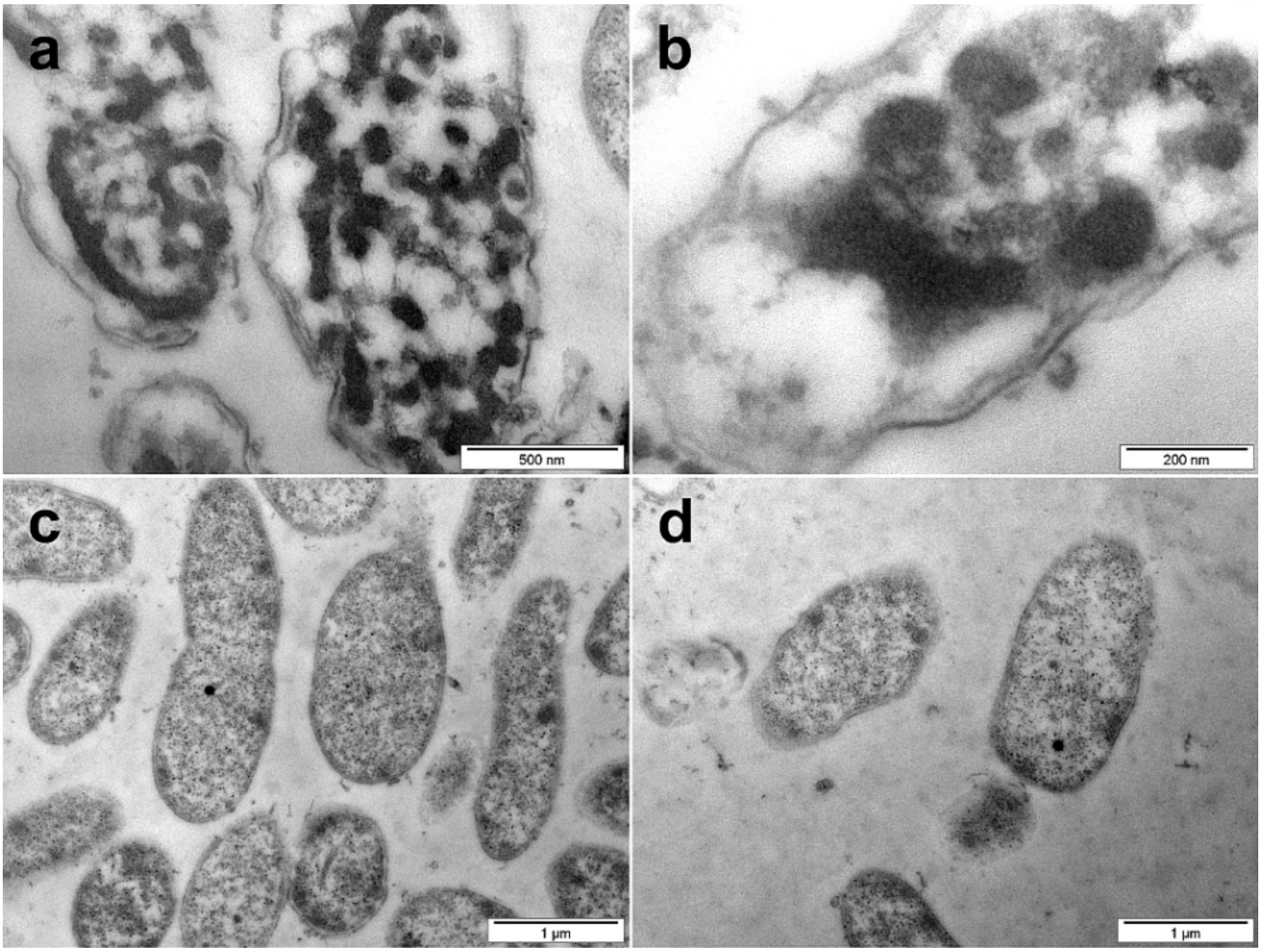

Supplementary Figure S2 I **TEM of HR 1-18-treated *P. aeruginosa*. a, b**, TEM of 6.25 x 10^7/ml *P. aeruginosa* ATCC 10145, in 10 mM NaP/ 0.25 % glucose/ pH 5.5, treated with 167 µg/mL HR1-18 ($HRNR_{2556-2677}$)(GRHGSGLGHSSSHGQHGSGSGR) for 1h at ambient temperature. **c, d**, control. Note the condensation of electron-dense cytoplasmic material in HR1-18-treated bacteria (**a, b**). Images are representative of two independent experiments, sampling on average 10 images.

Supplementary Figure S3

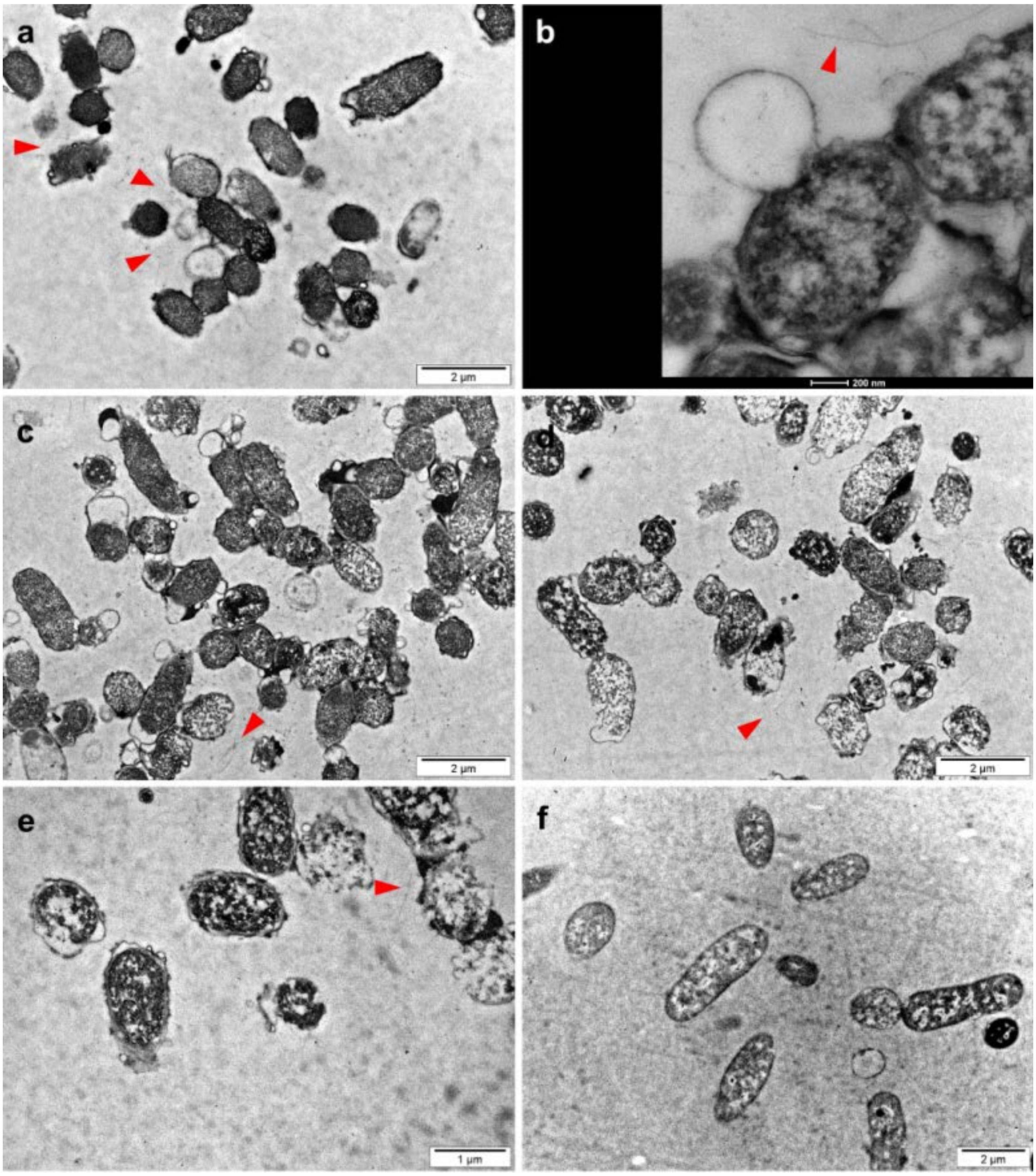

Supplementary Figure S3 I Ultrastructure kinetics in rSUMO3-HRNR$_{2591\text{-}2684}$-treated *E. coli*. TEM of 6.25 x 10^7/ml *E. coli* ATCC 11775, in 10 mM NaP/ 1% TSB/ pH 5.5, treated with 500 µg/mL rSUMO3-HRNR$_{2591\text{-}2684}$ for 5min (**a, b**), 20min (**c**), 1h (**d**) and 2h (**e**) at RT. **f**, buffer control (2h treatment). Note the absence of membrane perturbation and the presence of few blebs of the outer membrane with an occasional ballooning and some electron-dense cytoplasmic aggregates already after 5 min incubation. After 1h and 2h treatment intracytoplasmic electron-dense aggregates accumulate in rSUMO3-HRNR$_{2591\text{-}2684}$-treated bacteria (**d**, **e**). At higher magnification, in samples of rSUMO3-HRNR$_{2591\text{-}2684}$-treated bacteria often nanofiber-like structures were detected (**a** - **e**, red arrows). Images are representative of two independent experiments, sampling on average 10 images per condition in each experiment.

Supplementary Figure S4

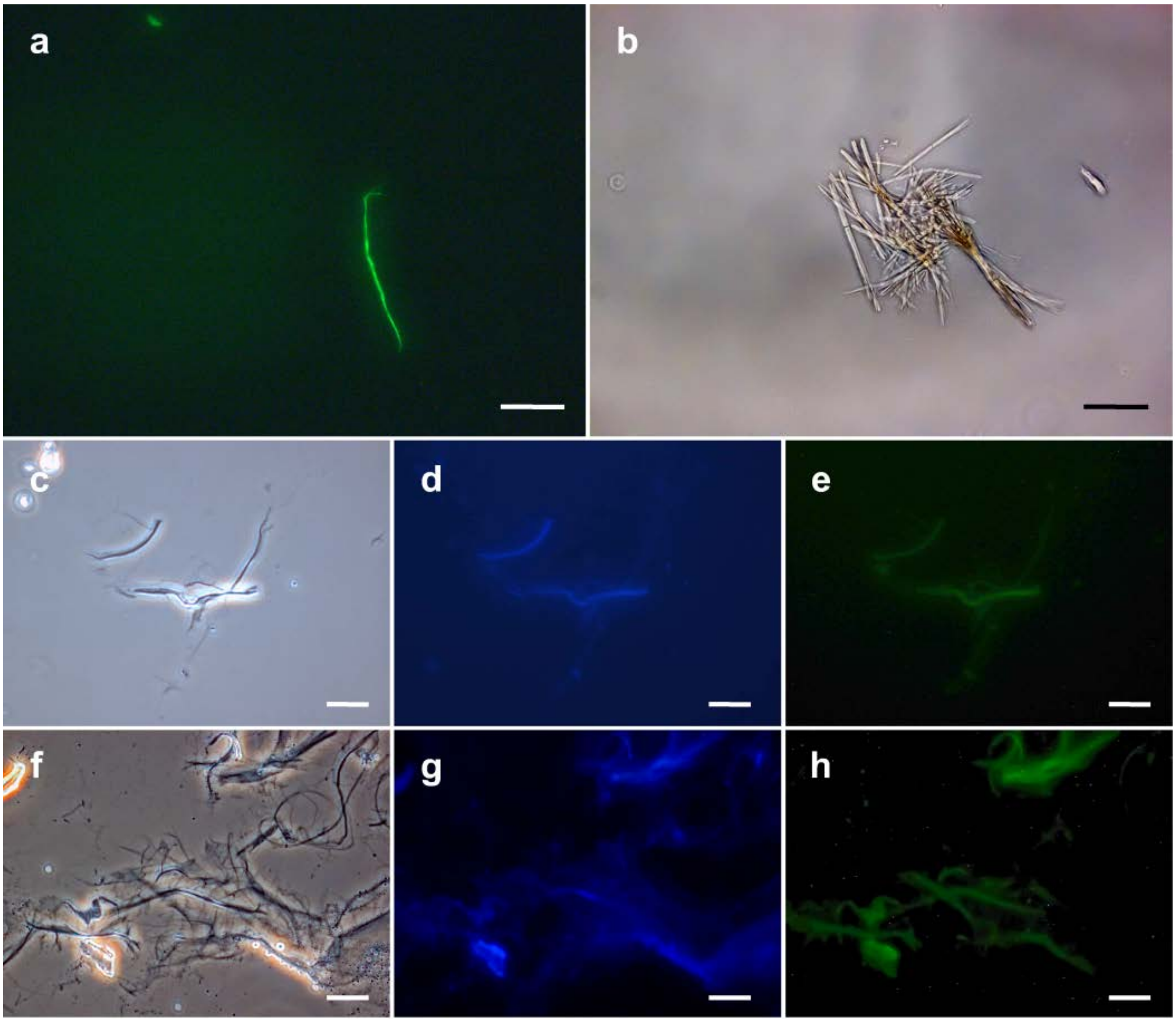

Supplementary Figure S4 I rSUMO3-HRNR$_{2591\text{-}2684}$ forms amyloid-like nanostructures.
rSUMO3-HRNR$_{2591\text{-}2684}$ was treated with ultrasound and then analyzed by monitoring thioflavin T fluorescence (**a**) and light microscopy (**b**) for amyloid-formation. In a second experimental series, FITC-labeled rSUMO3-HRNR$_{2591\text{-}2684}$ was treated with ultrasound and then monitored by light microscopy (**c, f**), for DAPI-staining (**d, g**) and for fluorescence, after storage for 1 h (**c** - **e**) and 20 h (**f** - **h**), respectively. Scale bar 20 µm.

Supplementary Figure S5

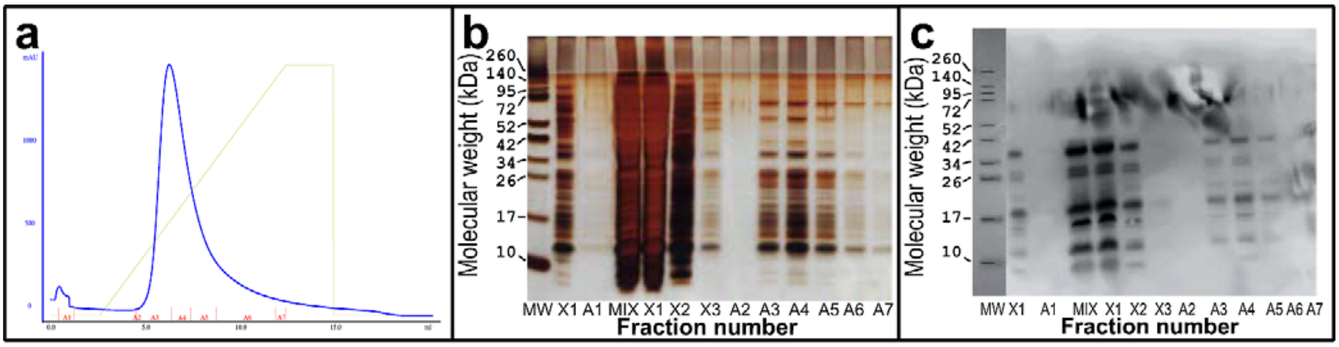

Supplementary Figure S5 I Identification of rSUMO3-HRNR$_{2591\text{-}2684}$-interacting proteins upon SulfoLink®–coupling resin-chromatography. An *E. coli*-extract was applied to a SulfoLink®–column and bound proteins were eluted with an increasing gradient of NH_4Cl-buffer (**a**). Aliquots of each fraction were analyzed by SDS-PAGE and silver staining (**b**) and by a Far-Western blot (**c**) using rSUMO3-HRNR$_{2591\text{-}2684}$ as "bait" protein and polyclonal antibodies against HRNR$_{2591\text{-}2684}$ for visualization of protein interaction. MW: molecular weight standards; MIX: crude *E. coli*-extract; X1 - X3: unbound material; A1 - A7: bound material.

Supplementary Figure S6

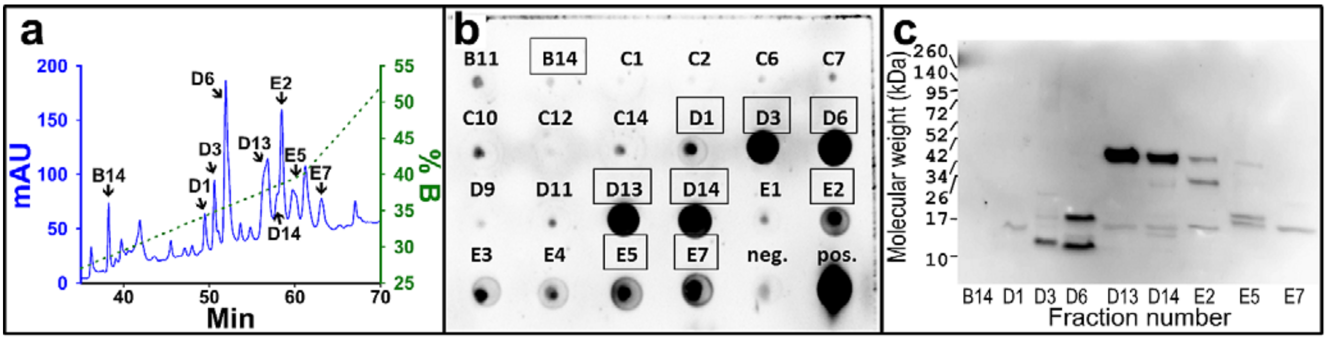

Supplementary Figure S6 I rSUMO3-HRNR$_{2591-2684}$ binds to *E. coli* ribosomal proteins. SulfoLink®–column-bound proteins of an *E. coli*-extract were separated on a **Jupiter® 300Å C18-RP-HPLC column** and eluted with an increasing gradient of **2-propanol (Prp) in aqueous 0.1 % TFA** (**a**). Aliquots of UV-absorbing peak fractions were analyzed in a Far- SUMO3-HRNR$_{2591-2684}$-dotblot analysis (**b**) and dotblot-positive fractions (highlighted as boxes) were subjected to a Far-rSUMO3-HRNR$_{2591-2684}$-Westernblot analysis (**c**). Note the presence of the most intensive band at 37 kDa (D13 and D14), and ~20 more or less intensive bands corresponding to a MW between 12 and 34 kDa.

Supplementary Figure S7a, b

a

Mascot Search Results

```
Database 1    : SwissProt 2014_08 (546238 sequences; 194363168 residues)
Database 2    : NCBInr 20140906 (48573147 sequences; 17378694133 residues)
Top Score     : 94 for 2::gi|485657623, 50S ribosomal protein L28 [Escherichia coli]
```

Mascot Score Histogram

Protein score is -10*Log(P), where P is the probability that the observed match is a random event.
Protein scores greater than 89 are significant (p<0.05).

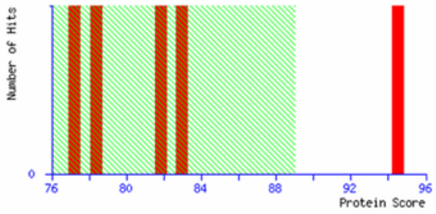

Protein sequence coverage: 90%

Matched peptides shown in red.

```
1    MYFWRIDMSR  VCQVTGKRPV  TGNNRSHALN  ATKRRFLPNL  HSHRFWVESE
51   KRFVTLRVSA  KGMRVIDKKG  IDTVLAELRA  RGEKY
```

Start-End	Observed	Mr(expt)	Mr(calc)	Delta	M	Peptide
1 - 10	1435.7560	1434.7487	1435.6377	-0.8889	1	-.MYFWRIDMSR.V + 2 Oxidation (M)
11 - 17	839.4370	838.4297	838.4371	-0.0074	0	R.VCQVTGK.R
18 - 25	913.4860	912.4787	912.4890	-0.0102	0	K.RPVTGNNR.S
26 - 33	841.4460	840.4387	840.4453	-0.0066	0	R.SHALNATK.R
36 - 44	1120.5880	1119.5807	1119.5938	-0.0130	0	R.FLPNLHSHR.F
45 - 51	924.4360	923.4287	923.4389	-0.0101	0	R.FWVESEK.R
45 - 52	1080.5380	1079.5307	1079.5400	-0.0093	1	R.FWVESEKR.F
52 - 57	791.4820	790.4747	790.4814	-0.0066	1	K.RFVTLR.V
53 - 57	635.3850	634.3777	634.3802	-0.0025	0	R.FVTLR.V
58 - 69	1330.7000	1329.6927	1330.7755	-1.0827	3	R.VSAKGMRVIDKK.G
62 - 69	946.4980	945.4907	945.5430	-0.0522	2	K.GMRVIDKK.G
65 - 69	601.3660	600.3587	601.3799	-1.0212	1	R.VIDKK.G
69 - 79	1214.6990	1213.6917	1213.7030	-0.0113	1	K.KGIDTVLAELR.A
70 - 79	1086.6070	1085.5997	1085.6081	-0.0083	0	K.GIDTVLAELR.A

b

Res#	Mass	Sequence	C-Term	Total Mass	Dev	S-S
9-85	8875.31	(M) SRVCQVTGKRPVTGNNR SHALNATKRRFLPNLHSHRF WVESEKRFVTLRVSAKGMRV IDKKGIDTVLAELRARGEKY (-)	-OH	8875.31	0.23	-

Supplementary Figure S7a, b I Identification of *E. coli* 50S ribosomal protein L28 in rSUMO3-HRNR$_{2591\text{-}2684}$-Far-Westernblot-positive HPLC fractions. In fraction **D3 (Supplementary Figure S6)** the *E. coli* 50S ribosomal protein L28 was identified (Mascot search reveals amino acid sequences of matching peptide fragments (**a, b**). MS-analysis of the D3 fraction before digestion revealed a major 8876,092 Da mass (average [Mr+H] mass) corresponding to an NH_2-terminally truncated version of L28 (residues 9-85).

Supplementary Figure S7c – g

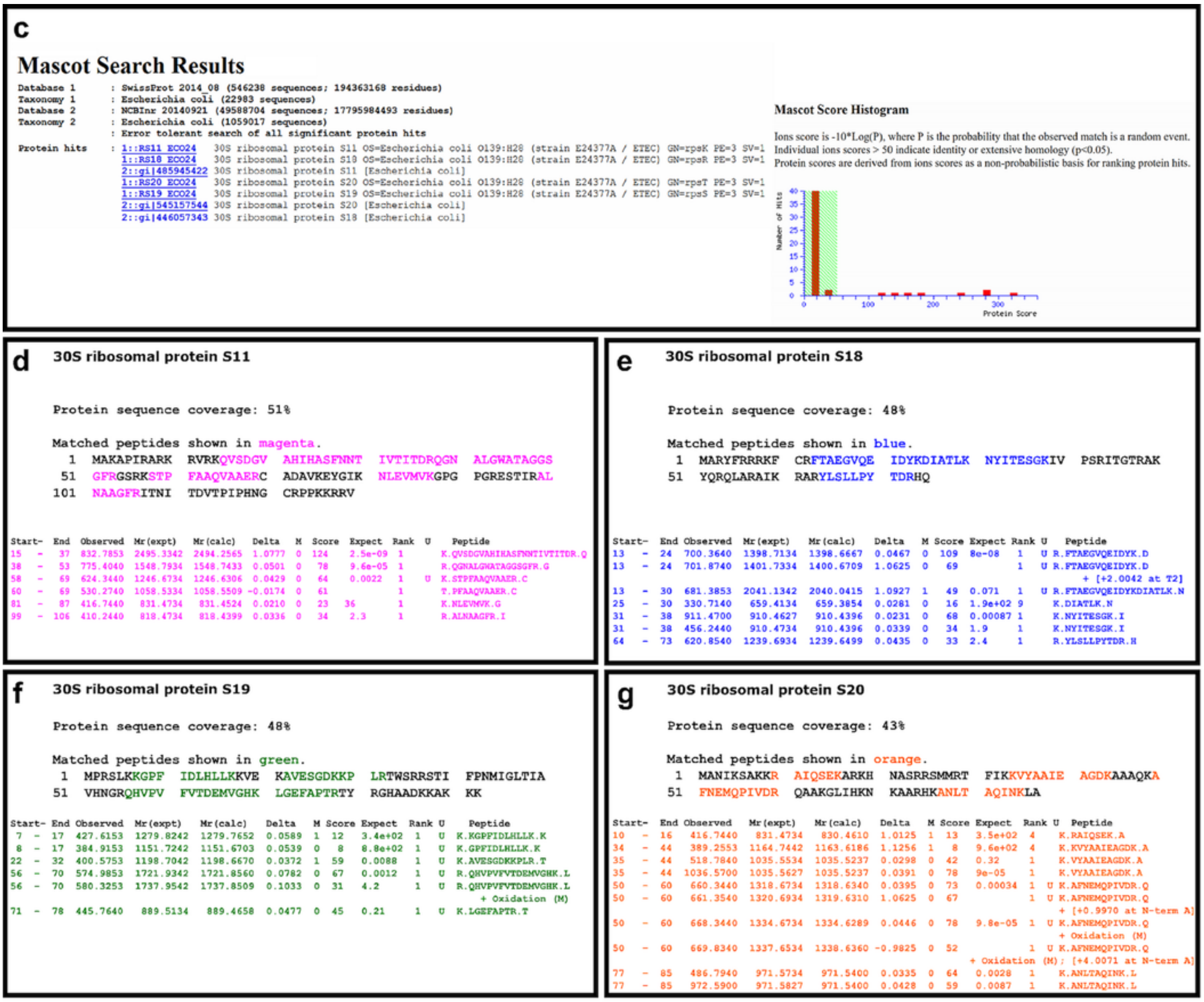

Supplementary Figure S7c-g I Identification of the *E. coli* 30S ribosomal proteins S11, S18, S19 and S20 in SUMO3-HRNR$_{2591-2684}$-Far-Westernblot-positive HPLC fractions. In fraction **D6 (Supplementary Figure S6)** the *E. coli* 30S ribosomal proteins S11, S18, S19 and S20 were identified. Amino acid sequences of identified peptide fragments from digested D6 fraction are shown separately for each 30S ribosomal protein using separate colors for different ribosomal proteins (**c - g**).

Supplementary Figure S7h, i

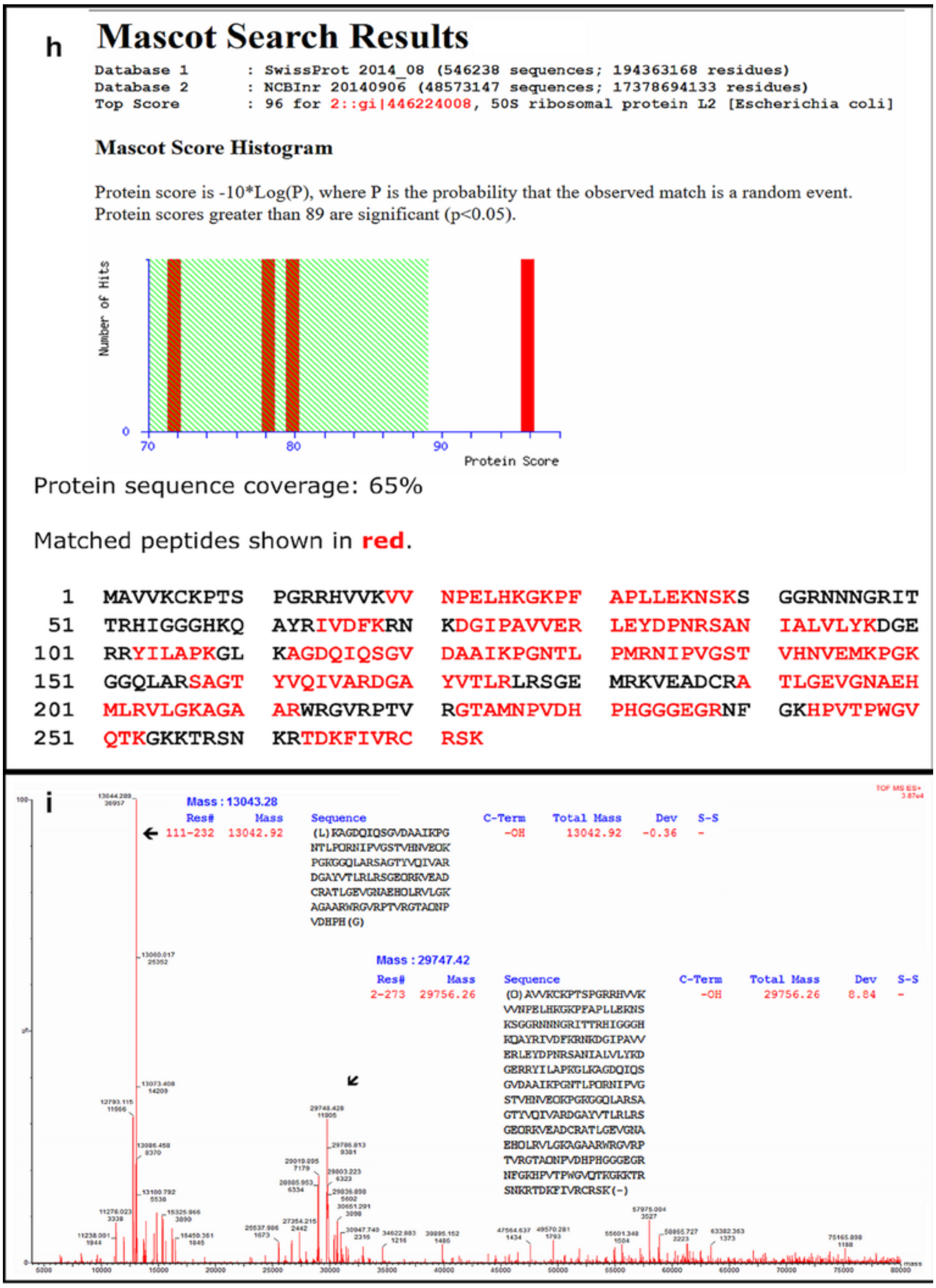

Supplementary Figure S7h, i I Identification of the 50S *E. coli* ribosomal protein L2 in rSUMO3-HRNR$_{2591\text{-}2684}$-Far-Westernblot-positive HPLC fractions. In fraction D13 (Supplementary Figure S6) the *E. coli* 50S ribosomal protein L2 was identified (**h, i**). MS-analysis of the D13 fraction before digestion revealed 2 major constituents (**i**). The 29748.428 da (average $[M_r{+}H]$) mass matches most closely to the calculated M_r mass for the entire L2 amino acid sequence lacking the aminoterminal methionine. The most intense 13044.289 da mass signal most probably represents an internal L2 fragment (**i**).

Supplementary Figure S8

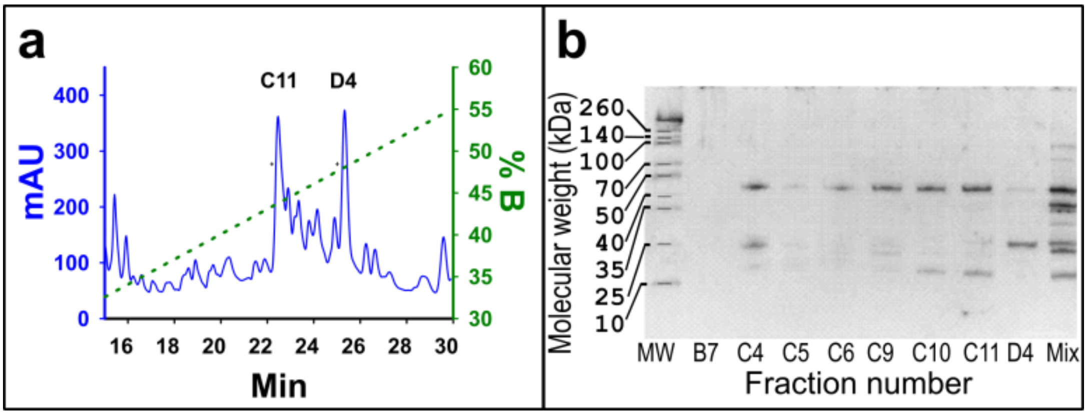

Supplementary Figure S8 I HRNR$_{2591\text{-}2684}$ binds to 30S and 50S *E. coli* ribosomal proteins. SulfoLink®-column-bound proteins of an *E. coli*-extract were separated on a **Jupiter® 300Å C18-RP-HPLC column** and were eluted with an increasing gradient of **acetonitrile (ACN) in aqueous 0.1 % TFA** (**a**). Aliquots of selected, in a HRNR-Far-Dotblot analysis positive HPLC fractions (C4 - D4) as well as SulfoLink®-column-bound proteins as control (Mix), were subjected to a HRNR-Far-Westernblot and analyzed with HRNR$_{2591\text{-}2684}$ antibodies (**b**). Some bands seen in the crude ribosome extract (mix) are missing in HPLC fractions (e.g. at 20, 24 and 25 kDa). MW: Molecular weight marker.

Supplementary Figure S9

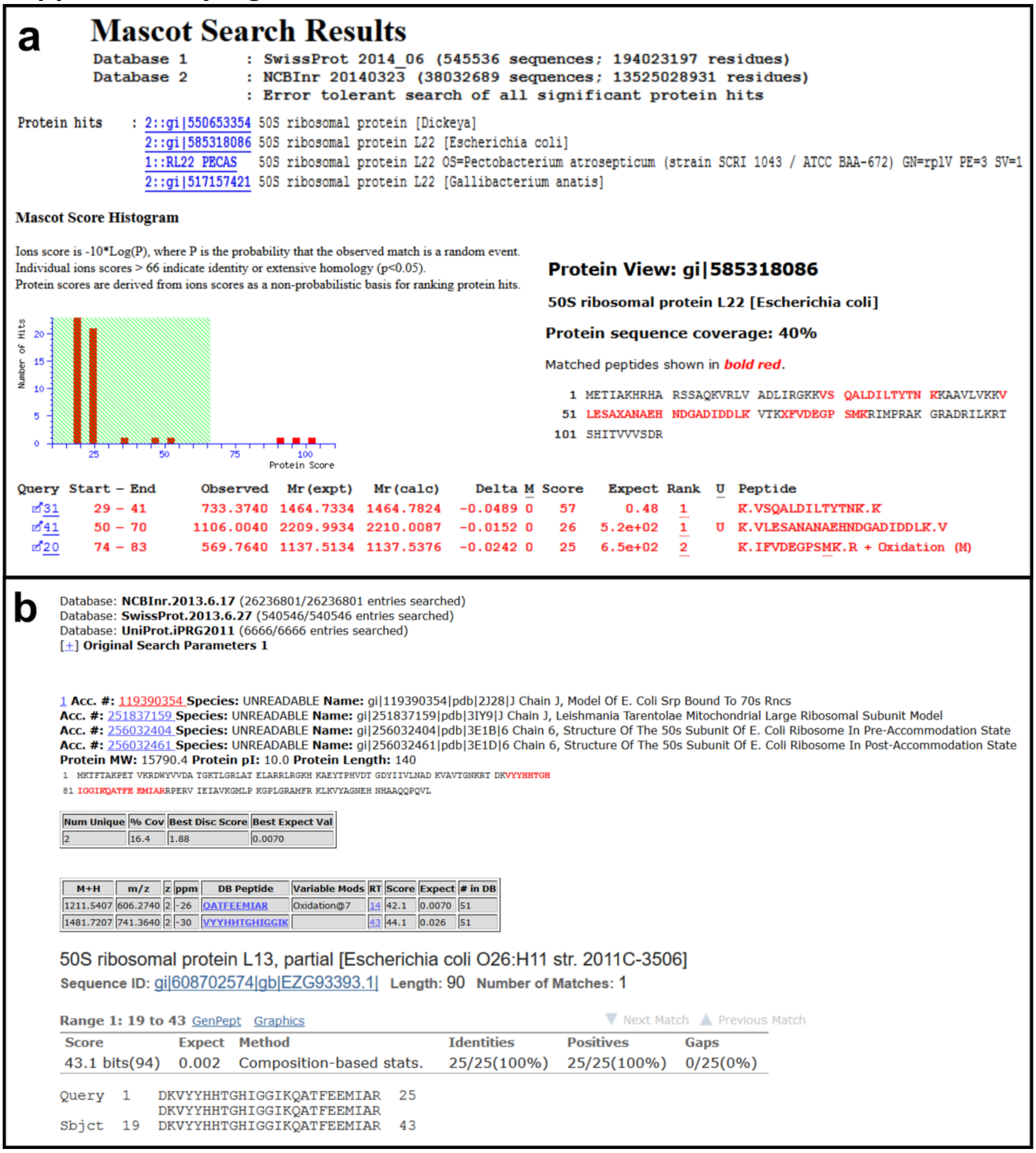

Supplementary Figure S9 I Identification of 50S *E. coli* ribosomal proteins L22 and L13 in HRNR$_{2591-2684}$-Far-Westernblot-positive HPLC fractions. SulfoLink®–column-bound proteins of an *E. coli*-extract, separated on a **Jupiter 300Å C18-RP-HPLC** column, eluted with **acetonitrile**, showing band(s) upon HRNR$_{2591-2684}$-Far-Westernblot (**Supplementary Figure S8**), were subjected to reduction, alkylation and tryptic digestion with subsequent MS/MS analysis at a QTOF-2 ESI-MS. Whereas in fraction **D4** the *E. coli* 50S ribosomal protein L22 was identified (**a**), MS/MS analyses revealed the *E. coli* 50S ribosomal protein L13 in fraction **C11** (**b**).

Supplementary Figure S10

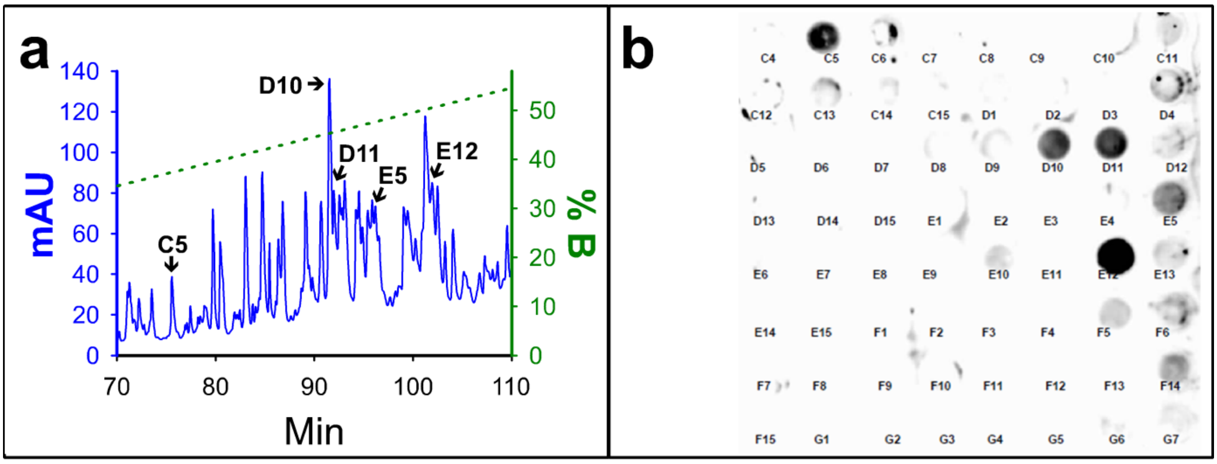

Supplementary Figure S10 I LC-MS/MS analyses identify a wide range of 50S and 30S *E. coli* ribosomal proteins with apparent HRNR-binding properties. SulfoLink®–column-bound proteins of an *E. coli*-extract were separated on a **Aeris® widepore RP-HPLC column** with a gradient of **Prp in aqueous 0.1 % TFA** (**a**) and fractions were analyzed for rSUMO3-HRNR$_{2591-2684}$-binding proteins in a HRNR-Far-dot blot system (**b**). Note strongest dots in fractions **C5, D10**, **D11**, **E5** and **E12**.

Supplementary Figure S11

Accession	Description	Score
W1G3Y5	30S ribosomal protein S10 OS=Escherichia coli ISC11 GN=rpsJ PE	22,27
W1G5N2	30S ribosomal protein S11 OS=Escherichia coli ISC11 GN=rpsK PE	12,95
W1FQT2	30S ribosomal protein S12 OS=Escherichia coli ISC11 GN=rpsL PE	16,63
W1FUI0	30S ribosomal protein S2 OS=Escherichia coli ISC11 GN=rpsB PE=	130,12
W1G3V3	30S ribosomal protein S3 OS=Escherichia coli ISC11 GN=rpsC PE=	121,77
W1G3T6	30S ribosomal protein S4 OS=Escherichia coli ISC11 GN=rpsD PE=	52,04
W1G3W9	30S ribosomal protein S5 OS=Escherichia coli ISC11 GN=rpsE PE=	60,37
W1G2U2	30S ribosomal protein S6 OS=Escherichia coli ISC11 GN=rpsF PE=	39,47
W1G213	50S ribosomal protein L10 OS=Escherichia coli ISC11 GN=rplJ PE=	35,14
W1G1R6	50S ribosomal protein L11 OS=Escherichia coli ISC11 GN=rplK PE=	19,38
W1G5Q1	50S ribosomal protein L14 OS=Escherichia coli ISC11 GN=rplN PE	8,93
W1G3U0	50S ribosomal protein L15 OS=Escherichia coli ISC11 GN=rplO PE	37,59
W1G7H8	50S ribosomal protein L17 OS=Escherichia coli ISC11 GN=rplQ PE	11,89
W1G7I8	50S ribosomal protein L18 OS=Escherichia coli ISC11 GN=rplR PE	7,37
W1G7K5	50S ribosomal protein L2 OS=Escherichia coli ISC11 GN=rplB PE=	23,78
W1G2X9	50S ribosomal protein L20 OS=Escherichia coli ISC11 GN=rplT PE	41,15
W1FUZ6	50S ribosomal protein L21 OS=Escherichia coli ISC11 PE=3 SV=1	24,46
W1G611	50S ribosomal protein L23 OS=Escherichia coli ISC11 GN=rplW PE	9,52
W1G3U9	50S ribosomal protein L24 OS=Escherichia coli ISC11 GN=rplX PE	12,00
W1FT83	50S ribosomal protein L3 glutamine methyltransferase OS=Escheri	14,93
W1G5R1	50S ribosomal protein L3 OS=Escherichia coli ISC11 GN=rplC PE=	37,65
W1G3V7	50S ribosomal protein L4 OS=Escherichia coli ISC11 GN=rplD PE=	220,74
W1G602	50S ribosomal protein L5 OS=Escherichia coli ISC11 GN=rplE PE=	95,61
W1G5Z8	50S ribosomal protein L6 OS=Escherichia coli ISC11 PE=3 SV=1 -	5,80
W1G0Z3	50S ribosomal protein L9 OS=Escherichia coli ISC11 GN=rplI PE=	123,48
W1G024	LSU ribosomal protein L1p (L10Ae) OS=Escherichia coli ISC11 PE=	25,49
W1G016	LSU ribosomal protein L7/L12 (P1/P2) OS=Escherichia coli ISC11	376,57
W1G3L1	Ribosomal protein OS=Escherichia coli ISC11 PE=3 SV=1 - [W1G3	29,47
W1FWK2	Ribosomal RNA small subunit methyltransferase C OS=Escherichia	12,87
W1FZR0	Ribosomal RNA small subunit methyltransferase E OS=Escherichia	5,62
W1FUM5	Ribosomal silencing factor RsfS OS=Escherichia coli ISC11 GN=rsf	92,69
W1FS83	SSU ribosomal protein S1p OS=Escherichia coli ISC11 PE=4 SV=1	294,85
W1FR69	SSU ribosomal protein S7p (S5e) OS=Escherichia coli ISC11 PE=4	14,31

Protein	Peptide sequence	Modification(s)
30S ribosomal pr	LVDIVEPTEKTVDALMR	
30S ribosomal pr	FTVLISPHVNKDAR	
30S ribosomal pr	FTVLISPHVNKDARDQYEIR	
30S ribosomal pr	LVDIVEPTEK	
30S ribosomal pr	ITNITDVTPIPHnGcRPPK	N13(Deamidated); C15(Pr
30S ribosomal pr	QGNALGWATAGGSGFR	
30S ribosomal pr	KSTPFAAQVAAER	
30S ribosomal pr	LTNGFEVTSYIGGEGHNLQEHSVILIR	
30S ribosomal pr	SNVPALEAcPQKR	C9(Propionamide)
30S ribosomal pr	GALDcSGVKDR	C5(Propionamide)
30S ribosomal pr	ELEKLENSLGGIKDmGGLPDALFVIDADHEHIAIK	M15(Oxidation)
30S ribosomal pr	ELEKLENSLGGIKDMGGLPDALFVIDADHEHIAIK	
30S ribosomal pr	LENSLGGIKDMGGLPDALFVIDADHEHIAIK	
30S ribosomal pr	DMGGLPDALFVIDADHEHIAIK	
30S ribosomal pr	EANNLGIPVFAIVDTNSDPDGVDFVIPGNDDAIR	
30S ribosomal pr	SQDLASQAEESFVEAE	
30S ribosomal pr	ELEKLENSLGGIK	
30S ribosomal pr	TVPMFNEALAELNKISAR	
30S ribosomal pr	GKILFVGTKR	
30S ribosomal pr	NKVHIINLEK	
30S ribosomal pr	TVPmFNEALAELNK	M4(Oxidation)
30S ribosomal pr	WLGGMLTNWK	
30S ribosomal pr	AGVHFGHQTR	

Supplementary Figure S11 | All-Fractions-Precursor-Ion-Area.

Supplementary Figure S12

Fraction	Protein	# Unique Peptides	Sequence Coverage (%)
C5	30S ribosomal protein S12 OS=Escheri	4	20,16%
	30S ribosomal protein S21 OS=Escheri	3	29,58%
	50S ribosomal protein L2 OS=Escherich	3	14,65%
D10	10 kDa chaperonin OS=Escherichia col	2	17,53%
	30S ribosomal protein S10 OS=Escheri	2	13,59%
	30S ribosomal protein S11 OS=Escheri	4	36,64%
	30S ribosomal protein S16 OS=Escheri	3	25,61%
	30S ribosomal protein S3 OS=Escherich	6	27,47%
	30S ribosomal protein S4 OS=Escherich	6	32,45%
	30S ribosomal protein S6 OS=Escherich	2	13,74%
	50S ribosomal protein L13 OS=Escheri	16	77,46%
	50S ribosomal protein L14 OS=Escheri	6	55,28%
	50S ribosomal protein L16 OS=Escheri	3	28,68%
	50S ribosomal protein L17 OS=Escheri	6	37,01%
	50S ribosomal protein L18 OS=Escheri	5	45,30%
	50S ribosomal protein L2 OS=Escherich	19	52,01%
	50S ribosomal protein L3 OS=Escherich	4	21,53%
	50S ribosomal protein L4 OS=Escherich	7	37,81%
	50S ribosomal protein L6 OS=Escherich	2	16,50%
	50S ribosomal protein L9 OS=Escherich	3	24,16%
	Cysteine desulfurase IscS OS=Escherich	2	7,67%
	DNA-binding protein OS=Escherichia c	4	23,36%
	DNA-directed RNA polymerase subuni	8	70,15%
	Ferredoxin, 2Fe-2S OS=Escherichia col	2	18,92%
	FKBP-type peptidyl-prolyl cis-trans iso	2	28,69%
	Pseudouridine synthase OS=Escherichi	5	22,13%
	Pseudouridine synthase OS=Escherichi	3	15,46%
	Ribosomal protein OS=Escherichia coli	2	17,99%
	Seryl-tRNA synthetase OS=Escherichia	2	15,18%
	Signal recognition particle protein OS=	2	5,74%
	Single-stranded DNA-binding protein (	2	13,04%
	SSU ribosomal protein S7p (S5e) OS=E	4	53,57%
	SSU ribosomal protein S8p (S15Ae) OS	2	22,83%
	Translation elongation factor Tu OS=E	2	19,40%
D11	30S ribosomal protein S3 OS=Escherich	2	16,74%
	30S ribosomal protein S4 OS=Escherich	2	13,25%
	50S ribosomal protein L13 OS=Escheri	10	48,59%
	50S ribosomal protein L14 OS=Escheri	3	44,72%
	50S ribosomal protein L18 OS=Escheri	5	56,41%
	50S ribosomal protein L2 OS=Escherich	4	23,08%
E5	30S ribosomal protein S10 OS=Escheri	3	21,36%
	30S ribosomal protein S3 OS=Escherich	10	48,50%
	30S ribosomal protein S4 OS=Escherich	4	37,09%
	30S ribosomal protein S6 OS=Escherich	8	67,18%
	50S ribosomal protein L14 OS=Escheri	2	30,08%
	50S ribosomal protein L22 OS=Escheri	3	35,45%
	50S ribosomal protein L23 OS=Escheri	4	39,00%
	50S ribosomal protein L5 OS=Escherich	3	18,44%
	50S ribosomal protein L9 OS=Escherich	2	22,15%
E12	30S ribosomal protein S2 OS=Escherich	2	19,92%
	30S ribosomal protein S3 OS=Escherich	4	34,76%
	30S ribosomal protein S4 OS=Escherich	2	23,84%
	30S ribosomal protein S6 OS=Escherich	10	73,28%
	50S ribosomal protein L14 OS=Escheri	2	30,08%
	50S ribosomal protein L5 OS=Escherich	5	34,64%
	50S ribosomal protein L9 OS=Escherich	3	27,52%
F14	30S ribosomal protein S10 OS=Escheri	4	35,92%
	30S ribosomal protein S11 OS=Escheri	3	36,64%
	30S ribosomal protein S12 OS=Escheri	3	41,13%
	30S ribosomal protein S2 OS=Escherich	13	60,59%
	30S ribosomal protein S3 OS=Escherich	13	56,65%
	30S ribosomal protein S4 OS=Escherich	6	44,37%
	30S ribosomal protein S5 OS=Escherich	4	34,13%
	30S ribosomal protein S6 OS=Escherich	4	32,82%
	50S ribosomal protein L10 OS=Escheri	6	38,79%
	50S ribosomal protein L11 OS=Escheri	4	33,80%
	50S ribosomal protein L14 OS=Escheri	2	30,08%
	50S ribosomal protein L15 OS=Escheri	8	59,03%
	50S ribosomal protein L17 OS=Escheri	4	26,77%
	50S ribosomal protein L18 OS=Escheri	2	17,95%
	50S ribosomal protein L2 OS=Escherich	3	15,38%
	50S ribosomal protein L20 OS=Escheri	6	30,51%
	50S ribosomal protein L21 OS=Escheri	4	56,25%
	50S ribosomal protein L23 OS=Escheri	3	33,00%
	50S ribosomal protein L24 OS=Escheri	3	39,42%
	50S ribosomal protein L3 glutamine m	3	8,71%
	50S ribosomal protein L3 OS=Escherich	6	35,89%
	50S ribosomal protein L4 OS=Escherich	18	79,60%
	50S ribosomal protein L5 OS=Escherich	9	44,13%
	50S ribosomal protein L6 OS=Escherich	2	9,71%
	50S ribosomal protein L9 OS=Escherich	10	63,76%

Supplementary Figure S12 I Data Base Research Results Edited.

Supplementary Tables

Supplementary Table S1 | Amino acid sequences of HRNR fragments generated as HRNR polypeptides or SUMO3-$(His)_6$-fusion proteins

$rHRNR_{1050-1172}$	THGQHGSTSGQSSSCGQHGASSGQSSSHGQHGSGSSQSSGYGRQGSGSGQSPGHGRGSGSRQSPSYGRHGSGSGRSSSSGQHGSGLGESSGFGHHES
$rHRNR_{2591-2684}$	SQHGSGSGHSSGYGQHGSRSGQSSRGERHGSSSGSSSHYGQHGSGSRQSSGHGRQGSGSGQSPSRGRHGSGYGHSSSHGQHGSGSGRSSSRGPY

Supplementary Table S2 I HRNR-primer-sequences used in this study

Name	Oligonucleotide-Sequence	A.T.1	A.T.2	vector
SUMO-hrnr-2-F	ACCCATGGGCAACACGGTTC	71,3°C		pET-Sumo
SUMO-hrnr-2-R	TCAAGACTCGTGGTGACCAAAGC	69,6°C		
pSu-hr3-b-2591-f (BsaI)	AAGGTCTCAAGGTAGTCAGCATGGGTCTGGCT	60°C	77,1°C	pSumo3
pSu-hr3-b-2684-r (BamHI)	AAGGATCCTTAATATGGGCCACGGCTGGAA	60°C	77,4°C	

A.T.1: Annealing temperature underlined sequence; A.T.2: Annealing temperature whole sequence. The complementary coding sequence is underlined.
